# M_1_-selective muscarinic allosteric modulation enhances cognitive flexibility and effective salience in nonhuman primates

**DOI:** 10.1073/pnas.2216792120

**Published:** 2023-04-27

**Authors:** Seyed A. Hassani, Adam Neumann, Jason Russell, Carrie K. Jones, Thilo Womelsdorf

**Affiliations:** ^a^Department of Psychology, Vanderbilt University, Nashville, TN 37240; ^b^Department of Pharmacology, Vanderbilt University, Nashville, TN 37240; ^c^Warren Center for Neuroscience Drug Discovery, Vanderbilt University, Nashville, TN 37240; ^d^Department of Biomedical Engineering, Vanderbilt University, Nashville, TN 37240

**Keywords:** acetylcholine, attention, donepezil, cognitive control, learning

## Abstract

Acetylcholine (ACh) in cortical neural circuits mediates how selective attention is sustained in the presence of distractors and how flexible cognition adjusts to changing task demands. The cognitive domains of attention and cognitive flexibility might be differentially supported by the M_1_ muscarinic acetylcholine receptor (mAChR) subtype. Understanding how M_1_ mAChR mechanisms support these cognitive subdomains is of highest importance for advancing novel drug treatments for conditions with altered attention and reduced cognitive control including Alzheimer’s disease or schizophrenia. Here, we tested this question by assessing how the subtype-selective M_1_ mAChR positive allosteric modulator (PAM) VU0453595 affects visual search and flexible reward learning in nonhuman primates. We found that allosteric potentiation of M_1_ mAChRs enhanced flexible learning performance by improving extradimensional set shifting, reducing latent inhibition from previously experienced distractors and reducing response perseveration in the absence of adverse side effects. These procognitive effects occurred in the absence of apparent changes of attentional performance during visual search. In contrast, nonselective ACh modulation using the acetylcholinesterase inhibitor (AChEI) donepezil improved attention during visual search at doses that did not alter cognitive flexibility and that already triggered gastrointestinal cholinergic side effects. These findings illustrate that M_1_ mAChR positive allosteric modulation enhances cognitive flexibility without affecting attentional filtering of distraction, consistent with M_1_ activity boosting the effective salience of relevant over irrelevant objects specifically during learning. These results suggest that M_1_ PAMs are versatile compounds for enhancing cognitive flexibility in disorders spanning schizophrenia and Alzheimer’s diseases.

## significance

Muscarinic receptors mediate the procognitive effects of acetylcholine, but it has remained unclear whether they differentially affect the cognitive subfunctions of attentional filtering, set shifting, and learning. To clarify the functional specificity of M_1_ mAChRs, we assessed these diverse functions using a recently developed, highly selective M_1_ PAM. This M_1_ PAM caused domain-specific cognitive improvement of flexible learning and extradimensional set shifting, reduced perseverations and enhanced target recognition during learning without altering attentional filtering functions. These domain-specific improvements contrasted to effects of a nonselective acetylcholinesterase inhibitor that primarily enhanced attention and caused dose-limiting adverse side effects. These results demonstrate domain-specific improvements in cognitive flexibility suggesting M_1_ PAMs are versatile compounds for treating cognitive deficits in schizophrenia and Alzheimer’s disease.

Cholinergic activity has far reaching consequences on attention and attentional control functions (1, 2) with long-standing suggestions that cholinergic modulation is involved in faster updating of expectations during learning (3–5). Depleting cholinergic innervation to the prefrontal cortex compromises while stimulation of cholinergic activity can enhance attentional control functions (6–10). These cholinergic effects have been suggested to be supported differently by nicotinic versus muscarinic receptors (11, 12). Antagonizing muscarinic cholinergic action with scopolamine in healthy humans and nonhuman primates (NHPs) increases false alarm rates and impairs sustained attention by slowing response times and impairing signal detection in two-alternative choice tasks (13–17). Consistent with these behavioral effects, neuronal recordings in the prefrontal cortex of NHPs have shown that attentional signaling depends on muscarinic receptor activation (18). One key open question from these insights is to what extent are attentional subcomponent processes supported by muscarinic signaling and whether there are subreceptors of the muscarinic receptor family that differentially support separable subcomponent processes underlying attention, such as filtering of distracting information, enhancing the flexible updating and shifting of attention sets, or supporting robust goal representations during goal-directed behavior. Each of these subcomponent processes has been associated in prior studies with the M_1_ mAChR, which is widely expressed in the cortex, striatum, and hippocampus (19, 20) and may thus mediate some of these muscarinic procognitive effects (2, 21).

One set of prior studies has implicated the M_1_ mAChR in memory processes because M_1_-selective ligands can restore deficits in novel object recognition (22, 23) and can partially reverse scopolamine-induced deficits in contextual fear conditioning consistent with M_1_-selective compounds enhancing the salience of the (aversive) outcomes during learning (22, 24, 25). But it has remained unclear whether the effects described in these studies are best accounted for by an improvement of memory, or whether enhanced cognitive control processes contribute to more effective encoding of stimuli as opposed to enhancing learning processes. A similar question about the specific cognitive process that is modulated arises when considering the M_1_ mAChR effects on different forms of attentional performance. While some studies have shown that M_1_ mAChR modulation is important for attentional modulation of neural firing (18, 26), behavioral studies using M_1_-selective PAMs in NHPs (27) and rodents (28) have not found primary improvements of sustained attention performance. Rather than modulating attention, the M_1_ mAChR actions improved behavior only in more demanding task conditions in which M_1_ modulation enhanced the adjustment of performance when task requirements changed (28). These results are consistent with findings showing that selective M_1_ mAChR ligands can facilitate the use of complex sensorimotor transformations to reach a goal (as in object-retrieval detour tasks) (29), and improve odor-based reversal learning of objects (30). These cognitive enhancing effects suggest that M_1_ mAChRs may be particularly important for higher cognitive control processes that go beyond attentional focusing or the filtering of distraction (2). However, it is not apparent which particular control processes might be supported by M_1_ mAChRs as the existing studies used widely varying tasks and a study using one of the classical cognitive control task (the antisaccade task) was not successful in identifying neural correlates of M_1_ mAChR–specific effects in the prefrontal cortex of NHPs (31).

The current study set out to address these questions about the specific procognitive role of the M_1_ mAChR in supporting attention and learning functions. Firstly, to distinguish cognitive subcomponent processes we devised two tasks. A visual search task allowed for distinguishing attentional subcomponent processes by varying distractor load and perceptual interference. And a intra-/extradimensional set-shifting learning task distinguishing cognitive control processes and cognitive flexibility. Secondly, we assessed NHP performance in these tasks using VU0453595, which is a recently developed subtype selective PAM for the M_1_ mAChR that does not activate the receptor directly but substantially potentiates the M_1_ mAChR response to endogenous ACh (22, 32, 33). This selective M_1_ PAM mechanism does not produce the dose limiting side effects which are prevalent with existing orthosteric mAChR agonists and AChEIs (34, 35), and which has the potential to treat deficits in attention control and cognitive rigidity prevalent in schizophrenia, Alzheimer’s disease, and substance use disorders (36–40). Assessing the procognitive profile of VU0453595 for these higher cognitive functions is therefore pivotal to advance therapeutic solutions for these widespread neuropsychiatric conditions (22, 41, 42).

We found that the M_1_ PAM VU0453595 exerts an inverted-U–shaped improvement of cognitive flexibility with increasing dose, causing faster learning, extradimensional set shifting, and reduced perseverations (i.e., enhanced flexibility), while leaving attentional filtering during visual search unaffected. These results are contrasted to the nonselective AChEI donepezil which improved attentional filtering with only marginal effects on cognitive flexibility (43).

## Results

We used four male rhesus macaques, with ages ranging from 7 to 11 y old, as subjects. Each of the four monkeys completed 60 sessions composed of 40 vehicle days and 7 d for each of the three tested doses of VU0453595 (0.3, 1, and 3 mg/kg). No dose-limiting adverse effects were observed in any of the 21 VU0453595 dosing days in any of the monkeys.

### M_1_ PAM VU0453595 Enhances Learning.

**Fig. 1.**
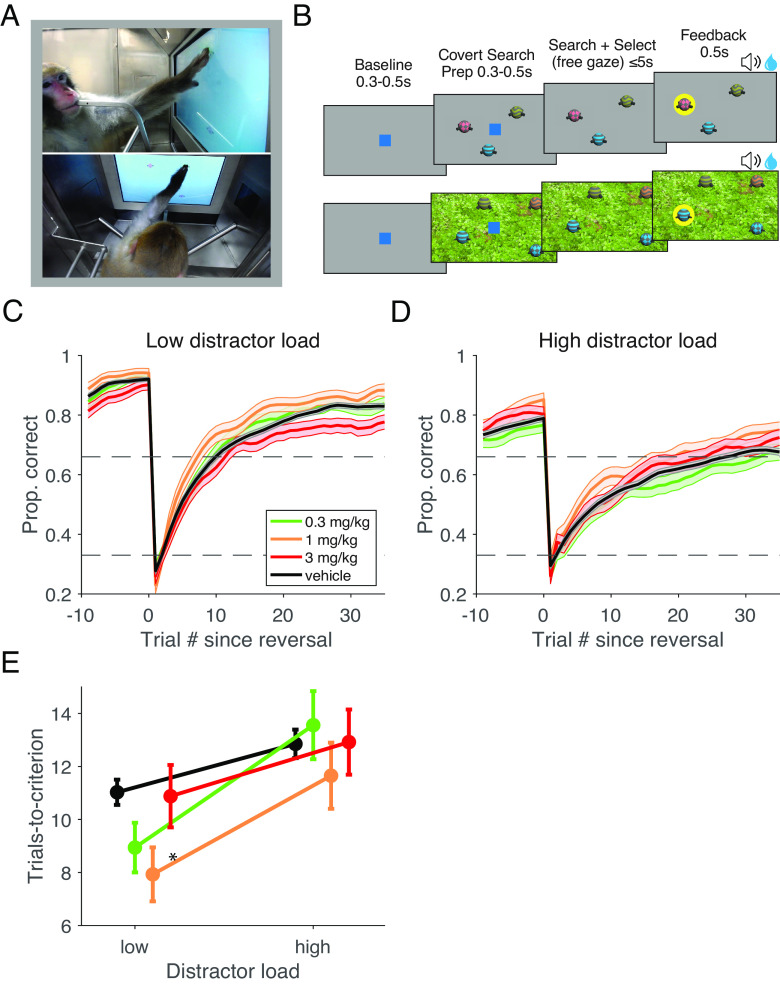
Task Design and Feature-Reward Learning Task Performance Enhancement by VU0453595 (*A*) Images of cage-mounted kiosk and monkey Ba utilizing the touch screen to perform the feature-reward learning (FRL) task taken via the video monitoring system. Both images are taken from the same time point from different angles. (*B*) Trial progression of the FRL task (*Top*) and the VS task (*Bottom*). The example FRL task here is a block with “high distractor load” where objects vary in both color and pattern. Each object contains only 1 feature from each feature dimension. Although the red checkered object was correctly chosen in this trial, the animal would need to learn through trial and error if the red or checkered feature was correct in order to optimally acquire reward from future objects in this block. The example VS block here shows a trial with three distractors and a target object that is defined by three features: blue, striped, and straight conical arms. The red distractor has zero features in common with the target, the yellow distractor has one feature in common with the target (striped pattern) and the blue distractor has two features in common with the target (blue color and straight conical arms). Trials in either task were initiated by a 0.3 to 0.5 s touch and hold of a central blue square (3° visual radius wide) after which the square disappears (for 0.3 to 0.5 s) and task objects (2.5° visual radius wide) are presented on screen. For the VS task, a structured background scene (here: a lawn) was used to distinguish the VS from the FRL task (which had a grey uniform background). For each visual search block, we drew a different random background scene from a set of five backgrounds independent of the target search feature. In either task, subjects have 5 s to select one of the objects with a 0.2 s touch and hold. Failure to choose an object resulted in an aborted trial which was ignored. Feedback for choice selection was provided 0.2 s after object selection for 0.5 s via both a visual halo around the chosen object as well as a auditory cue alongside any earned fluid. Both the frequency of the audio feedback and color of the feedback halo differed based on outcome. (*C*) Block-wise average learning curves for the low distractor load blocks of the FRL task aligned to block start for vehicle, 0.3, 1, and 3 mg/kg VU0453595, smoothed after the first three trials with a sliding window (shaded area: SE). Dotted horizontal lines signify 0.33 and 0.66 probabilities. (*D*) The same as *C* but for the high distractor load blocks. (*E*) Median trials-to-criterion, calculated as the first trial in a block that led to >70% performance over 10 subsequent trials, for the low and high distractor load blocks of the FRL task. For the low distractor load blocks, trials-to-criterion were 11.03 (SE: 0.38), 8.94 (SE: 0.75), 7.93 (SE: 0.81) and 10.88 (SE: 0.94) for vehicle, 0.3, 1, and 3 mg/kg doses of VU0453595, respectively. Only the 1 mg/kg dose was significantly different from vehicle [F(3,691) = 3.54, *P* = 0.01; η^2^ = .015; Tukey’s, *P* = 0.028; Cohen’s d = −0.352). For the high distractor load blocks, trials-to-criterion were 12.85 (SE: 0.43), 13.56 (SE: 1.03), 11.65 (SE: 1.00), and 12.92 (SE: 0.98) for vehicle, 0.3, 1, and 3 mg/kg doses of VU0453595, respectively, with no significant effect [F(3,565) = .40, n.s.].

Animals performed and consistently completed all 21 blocks of the feature learning task per session in all experimental conditions and expectedly showed faster learning in the easier low distractor load condition versus the more difficult high distractor load condition (Fig. 1 *C* and *D*). Administration of VU0453595 improved multiple measures of the learning performance compared to the vehicle control condition. In order to capture changes in learning speed, we calculated the trials-to-criterion as the first trial in a block that led to >70% accurate performance in the subsequent 10 trials. To reveal any temporally specific effects on learning, we implemented a linear mixed effects model on the median trials-to-criterion for the feature-reward learning (FRL) task (*SI Appendix*, *Supplemental Methods*). We found faster learning with 1 mg/kg dosing as evident in the early, middle, and last thirds of the 21 learning blocks per session with the first third of blocks showing the strongest effects (1 mg/kg, fixed effect: t(3674) = −2.67, *P* = 0.008; first third Cohen’s d = −0.228; overall Cohen’s d = −0.061) (*SI Appendix*, Fig. S1*A*). For this reason, all future analyses of the FRL task use the first third of blocks. Faster learning was particularly evident at low distractor load for which animals reached the trials-to-criterion at 7.93 (SE: 0.81) trials after a block switch with 1 mg/kg, compared to 11.03 (SE: 0.38) trials with vehicle [F(3,691) = 3.54, *P* = 0.01; η^2^ = .015; Tukey’s, *P* = 0.028; Cohen’s d = −0.352] (Fig. 1*E*). After the performance criterion was reached, VU0453595 also enhanced plateau performance (*SI Appendix*, Fig. S1*B*) and increased the proportion of blocks in which the animals reached the learning criterion at the 1 mg/kg dose (*SI Appendix*, Fig. S1*C*).

**Fig. 2.**
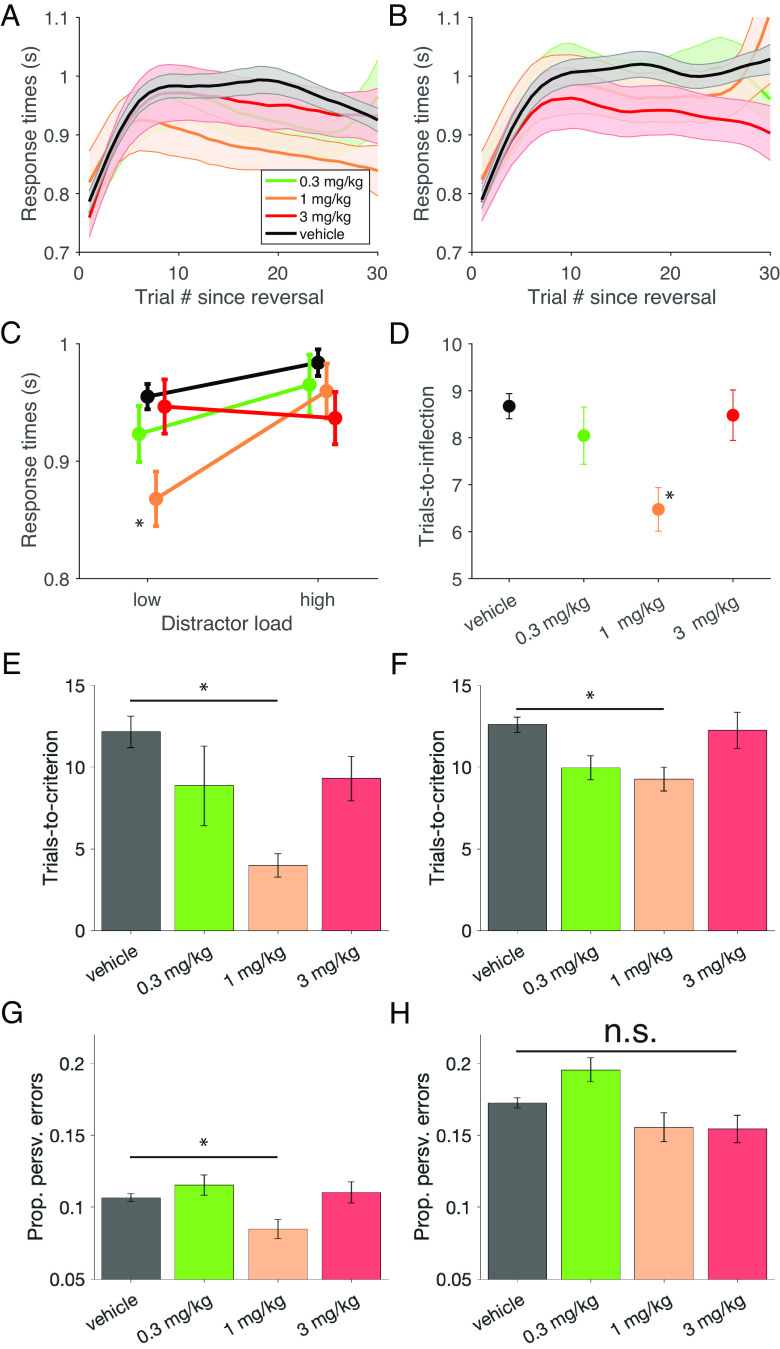
Feature-Reward Learning Task Efficiency and cognitive flexibility improvements with VU0453595 (*A*) The average RT curve of each session (correct trials only) aligned to block start for the low distractor load blocks of the FRL task for vehicle, 0.3, 1 and 3 mg/kg doses of VU0453595 (shaded area: SE) (*B*) The same as A but for high distractor load blocks of the FRL task. (*C*) Block-wise averages of the traces plotted in *A* and *B* visualized to compare RTs between distractor load conditions. Low distractor load blocks had RTs of 960 ms (SE: 11), 923 ms (SE: 24), 870 ms (SE: 23) and 974 ms (SE: 23) for vehicle, 0.3, 1, and 3 mg/kg doses of VU0453595, respectively. High distractor load blocks had RTs of 984 ms (SE: 11), 965 ms (SE: 26), 960 ms (SE: 23), and 937 ms (SE: 22). Only the 1 mg/kg dose of VU0453595 was significantly different from vehicle [F(3,1672) = 2.97, *P* = 0.03; η^2^ = 0.005; Tukey’s, *P* = 0.04; Cohen’s d = −0.350] (*D*) Trials-to-inflection for RTs in the low distractor load blocks defined as the first trial per block (excluding trial 2) that RTs become faster (error bars: SE). Trials-to-inflection was 8.7 (SE: 0.3), 8.0 (SE: 0.6), 6.5 (SE: 0.5) and 8.5 (SE: 0.5) for vehicle, 0.3, 1, and 3 mg/kg doses of VU0453595, respectively. Only the 1 mg/kg dose was significantly different from vehicle [F(3,193) = 2.68, *P* < 0.05; η^2^ = .040; Tukey’s, *P* = 0.017; Cohen’s d = −0.674]. (*E*) Block-wise average trials-to-criterion after extradimensional shifts were 12.2 (SE:1.0), 8.9 (SE: 2.4), 4.0 (SE: 0.7) and 9.3 (SE: 1.4) for vehicle, 0.3, 1, and 3 mg/kg doses of VU0453595, respectively. Only the 1 mg/kg dose showed a significant difference from vehicle [F(3,122) = 3.15, *P* = 0.03; η^2^ = 0.072; Tukey’s, *P* = 0.02; Cohen’s d = −0.868]. (*F*) Block-wise average trials-to-criterion after intradimensional shifts were 12.6 (SE: 0.5), 10.0 (SE: 0.7), 9.3 (SE: 0.7) and 12.3 (SE: 1.1) for vehicle, 0.3, 1, and 3 mg/kg doses of VU0453595, respectively. Only the 1 mg/kg dose showed a significant difference from vehicle [F(3,518) = 3.26, *P* = 0.02; η^2^ = .019; Tukey’s, *P* = 0.04; Cohen’s d = −0.349]. (*G*) The proportion of errors that were perseverative in nature with the feature that was perseverated being from the same feature dimension as the target feature. The proportion of perseverative errors from the target feature dimension were 10.7% (SE: 0.2), 11.5% (SE: 0.7), 8.5% (SE: 0.6) and 11.0% (SE: 0.7) for vehicle, 0.3, 1, and 3 mg/kg doses of VU0453595, respectively, with only the 1 mg/kg dose being significantly different from vehicle [F(3,1679) = 3.74, *P* = 0.01; η^2^ = 0.007; Tukey’s, *P* = 0.01; Cohen’s d = −0.243]. (*H*) The same as *G* but with the feature that was perseverated being from the distracting feature dimension (different from the target feature dimension). Proportions of perseverative errors from the distracting feature dimension were 17.3% (SE: 0.3), 19.6% (SE: 0.8), 15.6% (SE: 1.0) and 15.6% (SE: 1.0) for vehicle, 0.3, 1, and 3 mg/kg doses of VU0453595, respectively. There was a nonsignificant trend for a main effect of experimental condition [F(3,844) = 2.36, *P* = 0.07; η^2^ = 0.008].

Faster learning and improved performance accuracy in the 1 mg/kg dose condition were accompanied by faster response times (RTs). Over the course of a learning block, subjects showed a characteristic change of RTs with fast RTs early in the block that slowed down until an inflection point around the trial within the block when animals began to more consistently choose the rewarded target feature (Fig. 2 *A* and *B*). Notably, administering the middle (1 mg/kg) dose of VU0453595 led to significantly faster RTs of 870 ms (SE: 23 ms; low load) and 960 ms (SE: 23 ms; high load) in the low- and high-load conditions relative to vehicle RTs of 960 ms (SE: 11 ms; low load) and 984 ms (SE: 11 ms; high load) [F(3,1672) = 2.97, *P* = 0.03; η^2^ = 0.005; Tukey’s, *P* = 0.04; Cohen’s d = −0.350] (Fig. 2*C*). Moreover, the number of trials needed for the RTs to reach this inflection point was significantly fewer with the 1 mg/kg dose taking until trial 6.5 (SE: 0.5) relative to vehicle taking until trial 8.7 (SE: 0.3) [F(3,193) = 2.67, *P* < 0.05; η^2^ = 0.040; Tukey’s, *P* = 0.03; Cohen’s d = −0.674) (Fig. 2*D*).

### Improved Cognitive Control with M_1_ PAM VU0453595.

Learning a new feature-reward rule following a block switch entailed either identifying a target feature that was new or from a different feature dimension as in the previous block (extradimensional switches, ED), or from the same feature dimension as the previous target (intradimensional switches, ID). We found that the 1 mg/kg dose with VU0453595 significantly improved learning for both, ED and ID switches (Fig. 2 *E* and *F*) but not switches where the current target was from a novel feature dimension (data not shown). A large improvement was evident for ED switches with the average trials-to-criterion of 4.0 (SE: 0.7) after 1 mg/kg dose administration being significantly lower than the average 12.2 (SE: 1.0) trials-to-criterion of the vehicle condition [F(3,122) = 3.15, *P* = 0.03; η^2^ = .072; Tukey’s, *P* = 0.02; Cohen’s d = −0.868] (Fig. 2*E*). Please note that ED switches reported in our task were to a target of the previous distractor feature dimension and thus required disengaging from that dimension in addition to identifying the newly rewarded dimension. ID switches had a more moderate but still significant advantage after administration of 1 mg/kg dose of VU0453595 with a trials-to-criterion of 9.3 (SE: 0.7) relative to 12.6 (SE: 0.5) with vehicle [F(3,518) = 3.26, *P* = 0.02; η^2^ = 0.019; Tukey’s, *P* = 0.04; Cohen’s d = −0.349] (Fig. 2*F*).

The learning advantage after ED and ID switches indicates that VU0453595 at the 1 mg/kg dose enhanced cognitive control. Cognitive control also entails the ability to avoid erroneous perseverative responding. We quantified the perseverative responses as the proportion of repeated unrewarded choices to a feature in the target-feature dimension or in distractor-feature dimensions. We found that VU0453595 reduced perseverative responding to other features in the target feature dimension at 1 mg/kg from the 10.7% (SE: 0.2) of vehicle down to 8.5% (SE: 0.6) [F(3,1679) = 3.74, *P* = 0.01; η^2^ = 0.007; post hoc analysis of 1 mg/kg condition Tukey’s, *P* = 0.01; Cohen’s d = −0.243] (Fig. 2*G*). Perseverative responding to objects with features of the distractor dimension was moderately, but nonsignificantly reduced with VU0453595 [F(3,844) = 2.36, *P* = 0.07; η^2^ = 0.008] (Fig. 2*H*).

### M_1_ PAM VU0453595 Has No Consistent Effect on Interference Control.

Cholinergic compounds modulate attention and interference control (12, 43, 44). We evaluated these functions using a visual search (VS) task that varied the requirements to control interference from increasing numbers of distractor objects during search, and from increasing the number of features that were shared between target and distractors (target-distractor similarity, see *Methods and Materials*).

**Fig. 3:**
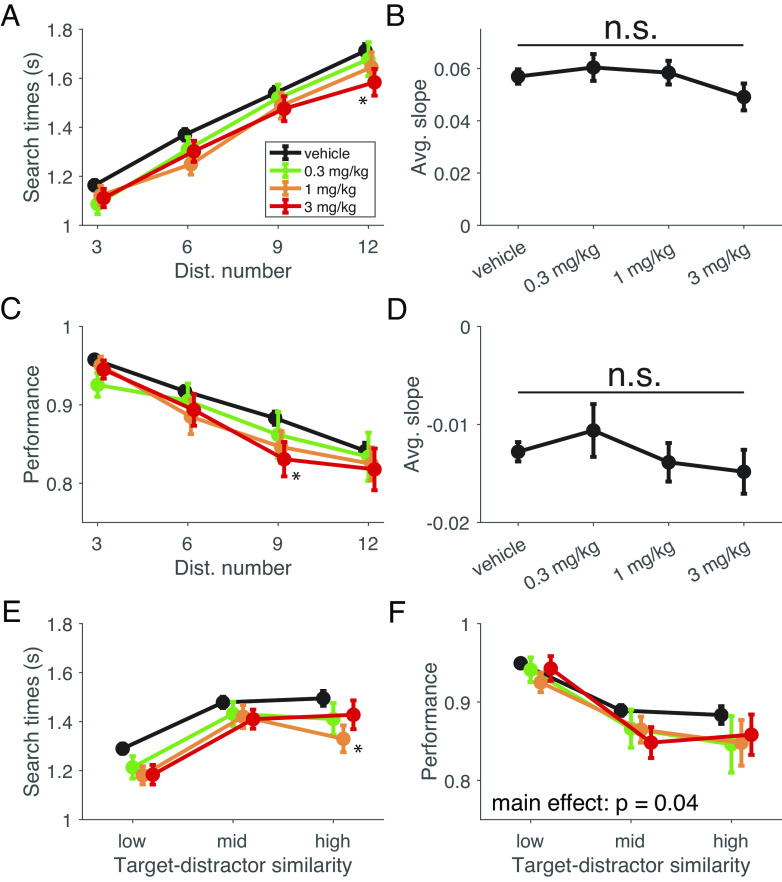
Distractor Effect and Interference Control Are Not Consistently Impacted by VU0453595 (*A*) Target detection durations (reaction times) as a function of distractor number for the second VS block. There was a significant main effect of experimental condition with a significant different between the 3 mg/kg dose of VU0453595 compared with vehicle [F(3,944) = 3.67, *P* = 0.01; η^2^ = .008; Tukey’s, *P* < 0.05]. The 3 mg/kg dose improved search times from 1.16 s (SE: 0.02), 1.37 s (SE: 0.02), 1.54 s (SE: 0.03) and 1.72 (SE: 0.03) with vehicle to 1.11 s (SE: 0.04), 1.30 s (SE: 0.04), 1.48 s (SE: 0.05) and 1.58 s (SE: 0.05) for 3, 6, 9, and 12 distractors, respectively. There was no significant change in the first VS block (data not shown). (*B*) The set size effect, operationalized as the slope of the linear fit of search times as a function of distractor numbers for the second VS block (0.057 (SE: 0.003), 0.060 (SE: 0.005), 0.058 (SE: 0.005) and 0.049 (SE: 0.005) for vehicle, 0.3, 1, and 3 mg/kg doses of VU0453595) was not significant [F(3,236) = 0.67, n.s.]. There was also no significant set size effect in the first VS block (data not shown). (*C*) VS task performance as a function of distractor number for the first VS block. There was a significant main effect of experimental condition with a significant different between the 3 mg/kg dose of VU0453595 compared with vehicle [F(3,944) = 3.80, *P* = 0.01; η^2^ = .010; Tukey’s, *P* = 0.04]. The 3 mg/kg dose reduced performance from 95.8% (SE: 0.4), 91.8% (SE: 0.7), 88.3% (SE: 0.8) and 84.1% (SE: 1.0) with vehicle to 94.5% (SE: 1.2), 89.4% (SE: 2.0), 83.1% (SE: 2.2) and 81.8% (SE: 2.7) for 3, 6, 9 and 12 distractors, respectively. There was no significant change in the second VS block (data not shown). (*D*) The set size effect, operationalized as the slope of the linear fit of performance as a function of distractor numbers for the first VS block [−0.013 (SE: 0.001), −0.011 (SE: 0.003), −0.014 (SE: 0.002) and −0.015 (SE: 0.002) for vehicle, 0.3, 1, and 3 mg/kg doses of VU0453595] was not significant [F(3,236) = 0.60, n.s.]. There was also no significant set size effect in the second VS block (data not shown). (*E*) VS task search times as a function of target-distractor similarity for the second VS block. There was a significant main effect of experimental condition with a significant different between the 1 mg/kg dose of VU0453595 compared with vehicle [F(3,708) = 4.67, *P* = 0.003; η^2^ = 0.018; Tukey’s, *P* = 0.02] but no significant set size effect [F(3,236) = 1.81, n.s.]. Search times were faster from 1.29 s (SE: 0.02), 1.48 s (SE: 0.02), and 1.49 (SE: 0.03) with vehicle to 1.18 s (SE: 0.04), 1.42 s (SE: 0.05) and 1.33 s (0.05) for low, medium, and high average target-distractor similarity, respectively. (*F*) VS task performance as a function of target-distractor similarity for the second VS block. There was a significant main effect of experimental condition but no significant post hoc comparison was found [F(3,708) = 2.84, *P* = 0.04; η^2^ = 0.011; Tukey’s, n.s.]. We also failed to find a significant set size effect [F(3,236) = 0.53, n.s.].

Animals showed prominent slowing of target detection times with increasing number of distractors from 3, 6, 9 to 12. VU0453595 did not consistently modulate this slowing with increasing distractor set size as evident by the similar slope of the linear fit across increasing numbers of distractor (Fig. 3 *A* and *B*). Similarly, the accuracy of target detection was not consistently affected by VU0453595 with no change of set size effects. For both, the raw values of target detection times and accuracy, some significant changes were observed (see Supplemental Results) but no systematic pattern could be extracted (Fig. 3 *C* and *D*). Similarly, VU0453595 did not consistently alter perceptual interference operationalized as changes in performance with increasing similarity between the target and distractors (Fig. 3 *E* and *F*). There were no changes in the set size effect for target detection times [first block: F(3,236) = 0.54, n.s.; second block: F(3,236) = 1.81, n.s.; Fig. 3*E*] or accuracy [first block: F(3,236) = 0.53, n.s.; second block: F(3,236) = 0.51, n.s.; Fig. 3*F*]. Similar to the distractor effect, the comparisons of how perceptual interference impacted raw target detection times and performance showed no systematic improvements (*SI Appendix*, *Supplemental Results*). We also looked at the relationship between search times and performance in both VS blocks independently and found no significant change in relationship at any of the tested doses (n.s.; fisher r to z transformation; *SI Appendix*, Fig. S2). No changes to speed of processing, operationalized as the time to response during familiarization trials (*Methods and Materials*) were observed with VU0453595 at any dose for neither the first VS block [F(3,236) = 0.56, n.s.] nor the second block [F(3,236) = 0.35, n.s.] (*SI Appendix*, Fig. S3).

### Comparison of VU0453595 Effects with the Literature and Consistency of Effects across Monkeys.

To evaluate how our study compared to previous studies, we surveyed 20 studies in NHPs (six using M_1_-selective PAMs) and thirty-two studies in rodents ((28) using M_1_-selective PAMs) and summarize them in Table 1 (study details are described in *SI Appendix*, Tables S2 and S3 for NHP and rodent studies, respectively). This survey highlighted two main ways the current study is distinct from existing studies beyond the use of the compound VU0453595.

**Table 1.** Summary of studies in NHPs and rodents assessing cognitive and behavioral effects using M_1_ PAMs specifically. For details about the individual NHP studies, see *SI Appendix*, Table S2 and for individual rodent studies, see *SI Appendix*, Table S3

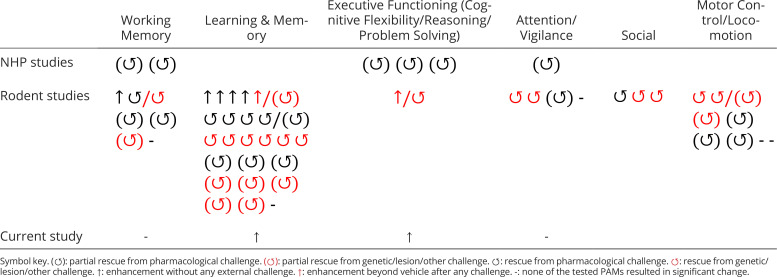

First, the type and number of tasks used here differ from typical behavioral assays. None of the surveyed nonhuman primate studies used a set-shifting task or varied the number and similarity of distractors to a target stimulus in an attention task. Of the six studies using M_1_ PAM compounds (PQCA, TAK-071, MK-7622, or VU0453595), two contained no behavior, four tested cognitive effects including problem solving (3/4), working memory (2/4), and vigilance/attention (1/4). Critically, this study shows cognitive enhancement relative to vehicle and does not involve reversing pharmacological challenge-mediated deficits in cognition unlike the other M_1_ PAM NHP studies surveyed here. Similarly, of the 28 rodent studies using M_1_ PAMs (VU compounds: 9/28; BQCA/PQCA: 9/28 studies; PF compounds: 6/28 studies; TAK-071: 4/28; MK-7622: 3/28; Other: 5/28) in diverse pharmacological challenge paradigms and disease models (Table 1 and *SI Appendix*, Table S3). Across these rodent M_1_ PAM studies, none quantified ED/ID set shifting, while one reported reversal learning performance (30), alongside behavioral assays testing learning and memory (19/28), locomotion/ motor control (9/28), working memory (6/28), attention/vigilance (3/28), social behavior (3/28), misc. exec. function (2/28), or satiety/drug abuse (1/28).

**Fig. 4.**
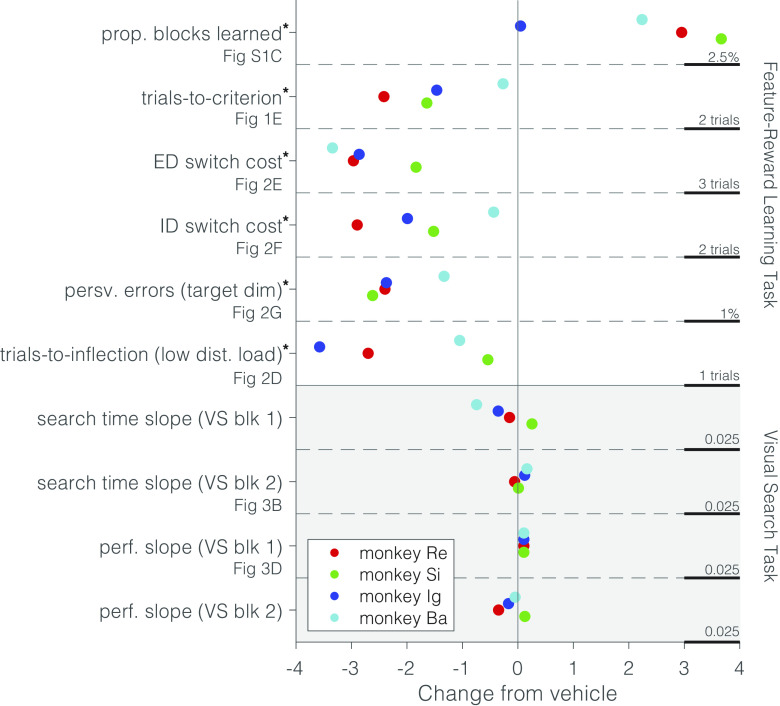
Results Summary and Consistency across Monkeys. Key results from the feature-reward learning task (*Top*) and the visual search task (*Bottom*). Measures and the respective figures showing each result (if applicable) are stated on the *y*-axis; asterisks indicate a significant effect for all monkeys combined. Values for each monkey represent the average change at the 1 mg/kg VU0453595 dose relative to vehicle scaled arbitrarily for each measure. The scaling for each measure is indicated on the right for 1 arbitrary unit along the *x*-axis. At the 1 mg/kg dose, VU0453595 enhances virtually all measures of the feature-reward learning task to some degree while no reliable changes in the visual search task were observed.

Second, our approach to test individual monkeys seven times per dose differs from all six previous NHP M_1_ PAM studies, which used one determination per dose per monkey. These previous studies focused analyses on the group level with on average eight NHPs used per dose (*SI Appendix*, Table S2), compared to four monkeys per dose in our study. This difference raises the question whether our study design succeeded to find consistent effects not only in individual subjects (which we aimed for by repeating each dose seven times), but also across subjects. We address this question by summarizing in Fig. 4 the average effect of 1 mg/kg VU0453595 relative to the vehicle condition for each subject (marked in different colors) and across all major performance metrics of the set-shifting task and the visual search task. The figure illustrates the consistency of the effects across monkeys and performance metrics, supporting the main conclusions of the previous sections that the M_1_ PAM enhanced metrics indexing cognitive flexibility (reduced switch costs, reduced perseverative errors and led to faster learning) with less consistent effects on metrics indexing distractor filtering.

## Double Dissociation of VU0453595 and Donepezil for Cognitive Flexibility and Interference Control

VU0453595 improved learning and reduced perseveration, but without reducing interference from distracting objects and features. This pattern of results contrasts to the effects of nonselective AChEI donepezil for which a prior study using the same tasks as in the current study found that an optimal dose range improved VS performance but without affecting reward learning and perseveration (43). To quantify this difference, we re-analyzed the performance of reward learning and visual search with donepezil in the prior study using the best dose for VS improvements (0.3 mg/kg) (43). This comparative approach revealed a double dissociation (Table 2). VU0453595 enhanced metrics of learning efficiency and cognitive flexibility but not metrics of interference control during VS, while donepezil made the animals more robust against distraction (improved interference control) during visual search but did so without improving feature-reward learning performance. Furthermore, at this dose, donepezil slowed down response times in the FRL task as well as search times in the VS task and even slowed the speed of processing early, partially as a consequence of dose-limiting side effects that accompanied donepezil. In contrast, VU0453595 at 1 mg/kg sped up response times in the FRL task without slowing down VS search times or the speed of processing and without any observable side effects (Table 2).

**Table 2.** Comparison of performance metrics with the best doses of VU0453595 and Donepezil

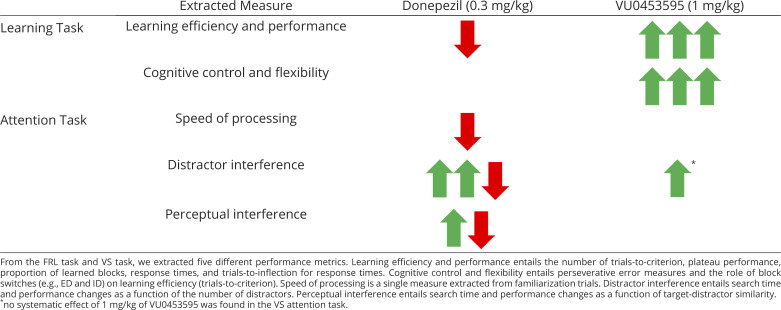

## Discussion

Here, we found that healthy adult rhesus monkeys show M_1_ mAChR–specific improvements of cognitive flexibility in a feature-reward learning task, while leaving attentional filtering unaffected. In particular, the middle of three doses of the M_1_ PAM VU0453595 increased the speed of learning a new feature-reward rule, particularly with extradimensional rule changes. At the same dose animals showed less perseveration on unrewarded features. These procognitive effects contrasted to the absence of consistent distractor-dependent changes in accuracy or search times during VS. Although we did find significantly faster search times in the second VS block only and a significant reduction in performance in the first VS block only (both at 3 mg/kg), no attentional effect, i.e., shift in the slope of performance or search times dependent on the number of distractors or on target-distractor similarity was found. At the dose range tested no adverse side effects were noted. This result pattern contrasts with the effects of donepezil which improved attentional filtering during VS at a dose at which it did not affect cognitive flexibility, but already resulted in dose-limiting side effects. Taken together, these findings document a functional dissociation of the role of M_1_ mAChR modulation with highly selective M_1_ PAMs, suggesting it is a versatile treatment target for disorders suffering from inflexible, rigid cognition and behavior including schizophrenia, Alzheimer’s disease, and addiction.

### M_1_ PAM Enhances Learning and Extradimensional shifts.

We found that the medium dose of VU0453595 improved learning of feature values. Compared to the vehicle control, the medium dose allowed subjects to reach the performance criterion 3.10 trials earlier at the low distractor load condition (Fig. 1*E*) and the number of trials to reach RT inflection decreased by 2.20 trials for low distractor load blocks (Fig. 2*D*) (see *SI Appendix*, *Supplemental Discussion* with regard to dose specificity of the effects). The learning improvement was particularly apparent with extradimensional (ED) switches, i.e., when the target feature in a block was from a different feature dimension as the target in the preceding block (Fig. 2*E*). Typically, ED switches take longer and are more difficult than intradimensional switches by requiring the recognition of a new dimension and integrating it in a new attentional set (45), suggesting that VU0453595 particularly benefits the flexible updating and switching of attention sets. This finding in NHPs extends the insights that the M_1_-selective ago-PAM BQCA can restore odor-based reversal learning of objects in transgenic mice (30).

Computationally, human and animal studies support the suggestion that an M_1_ PAM mechanism might enhance the updating of attention sets. Enhanced learning following ED switches in our task paradigm suggests that the M_1_ PAM VU0453595 allowed the animals to more effectively recognize that previously unrewarded, distracting, features became rewarded. The effect of VU0453595 is therefore akin to increasing the effective salience of those targets that were “learned distractors” from the previous block while suppressing the salience of current distractor features (Fig. 1). Recent modeling suggests that increasing effective salience is achieved with an attention–augmentation mechanism that enhances learning from attended features by actively unlearning (forgetting) values of unattended features (46). Various studies have documented that such an attention–augmentation mechanism is important for fast learning in complex tasks like the one used here (46–51). The effect of VU0453595 may thus enhance the effective salience of target features, consistent with neuronal recordings that show M_1_ mAChR activation in the prefrontal cortex is necessary during the early processing of targets (52). Support for this suggestion comes from an elegant multitask study in NHPs that found compromising muscarinic activity with scopolamine increased the proactive interference of prior spatial information onto current performance in a self-ordered search task (53). The current findings support the interpretation that potentiation of M_1_ mAChR activity reduces proactive interference with the net effect of enhanced effective salience.

Recent human studies found that the learning of stimulus-response reward probabilities is enhanced with the AChE inhibitor galantamine (5) and impaired when antagonizing muscarinic receptors with biperiden (54). In a Bayesian framework, these performance improvements were linked specifically to enhanced versus reduced weighting of top-down expectancies and prediction errors during learning (5, 54). In this framework, muscarinic receptor activity determined how fast prediction errors led to belief updating about how stimuli are linked to reward. The results of the current study is consistent with that framework by suggesting that enhanced belief updating and effective salience is mediated specifically through the potentiation of the M_1_ mAChR. Supporting this conclusion, in rodents, the M_1_-selective ago-PAM BQCA reverses scopolamine-induced deficits in a contextual fear conditioning consistent with M_1_ mAChR enhancing the salience of the (aversive) outcomes during learning (22, 24, 25).

These functions of the M_1_ mAChR may be realized in the prefrontal cortex. In primates, reversal learning and the extradimensional updating of attentional sets depend on dissociable subareas of the prefrontal cortex with ED shifting and the recognition of attention sets depending particularly on the ventrolateral prefrontal cortex (55, 56). Support for such a prefrontal mechanism comes from a rodent study that found the M_1_-selective PAM TAK-701 can partially reverse a deficit of target detection selectively on signal trials that followed no-signal trials when the deficit was induced by partially (~60%) depleting ACh afferents to the prefrontal cortex (28). These considerations support the notion that M_1_ mAChRs in prefrontal cortex are pivotal for the improved updating of attentional sets (2).

In previous work, faster learners of feature-reward associations were shown to have improved working memory capacity (46), which raises the possibility that M_1_ mAChR allosteric modulation may have affected learning through enhanced short-term memory of targets. We believe this is unlikely. While the nonselective muscarinic antagonist scopolamine impairs short term memory retention and nonselective AChE inhibitors partially reverse the deficit (57–61), the short-term deficits can be independent of the delay and more prominent for short or intermediate delays, making it unlikely that muscarinic receptors have primary effects on recurrent persistent delay representations (53, 59, 62, 63).

### M_1_ PAM Reduces Perseverative Responding.

A second main result of the current study is VU0453595 reduced response perseveration, allowing animals to avoid repeating erroneous responses to objects with the same nonrewarded features (Fig. 2*E*). This finding supports early insights into the effects of the muscarinic antagonist scopolamine in the prefrontal cortex to increase omissions (64), suggesting that it is the M_1_ mAChR that is particularly important for minimizing error rates. Support for the M_1_ specificity of these effects also comes from a study treating transgenic mice with an M_1_-selective ago-PAM which resulted in reduced erroneous choices of compound object discrimination in the trials after reversing object-reward associations (30).

Perseverative responding is the key characteristic of inflexible, habitual responding because it reflects that performance feedback is not utilized for adjusting behavior. It has been shown that performance feedback triggers transient activation of cholinergic neurons in the basal forebrain in mice (65) and activates the basal forebrain in humans (66). In the prefrontal cortex, cholinergic transients trigger gamma activity (67) that depends specifically on local M_1_ mAChRs (52), and thus could be a mechanism underlying improved recognizing feedback that leads to avoiding perseveration in our study.

Taken together, the reduction of perseverative responding with VU0453595 implicates the M_1_ mAChRs in the effective processing of feedback to adjust future performance. Perseverative, habitual responding is a hallmark of multiple psychiatric disorders including schizophrenia, obsessive compulsive disorder and substance use disorders (68, 69). The current result therefore bears particular relevance by suggesting that potentiating the M_1_ mAChR critically reduces perseverative response tendencies (70).

### M_1_ PAM Has No Consistent Effect on Interference Control over Distractors.

We found that VU0453595 did not affect VS performance differently with few or many distractors. Target detection response times were moderately faster and accuracy was moderately lower to a similar extent for 3, 6, 9, or 12 distractors (Fig. 3 *A*–*D*). This finding shows that the VU0453595 dose range that improved cognitive flexibility did not alter attentional filtering of distracting information. This finding adds clarity to diverse results in previous studies. Firstly, the absence of M_1_-specific distractor effects resonates with a recent finding in rodents that the M_1_-selective PAM, TAK-071, did not modulate the distracting effects of light on/off switches during a sustained attention task, but started to improve performance in the second half of testing when distraction ended, and the animals adjusted to a no-distractor regime (28). This result pattern is congruent with our result pattern. Allosteric modulation of the M_1_ mAChR improved adjusting behavior to challenges, but without improving interference control from distraction. A similar lack of effects of muscarinic modulation on distractor interference control were found in other task contexts. Scopolamine-induced deficits of continuous recognition performance can be partially reversed with an M_1_-selective agonist (71) or the nonselective muscarinic agonist milameline (72, 73), but this deficit reversal is independent of the similarity between distracting and target objects (27). Similarly, scopolamine does not alter distractor effects in an attentional flanker task, but rather causes an overall slowing and selective impairment of learning reminiscent of the reward learning effect we found (74).

The observed result pattern with the M_1_ PAM VU0453595 contrasts to apparent effects to reduce distraction with nicotinic modulation (12, 44, 75, 76), with nonselective cholinergic increases using donepezil (43) (Table 2), or with the improvement of target detection accuracy and visuospatial attentional orienting when enhancing cholinergic transmission from the basal forebrain (1, 77–79). Particularly relevant in this context is a prior NHP study that found the nicotinic alpha-4/beta-2 receptor agonist selectively enhanced distractor filtering when two stimuli underwent salient changes but had no effect on reversal learning speed (44).

One caveat when interpreting the absence of an effect with the M_1_ PAM VU0453595 is that it is unclear whether a higher dose of this ligand would have affected distractor filtering during VS performance. The highest dose used in this study (3 mg/kg, oral) is a magnitude lower than the maximum 30 mg/kg doses that previous studies found to have only mild adverse side effects (80), suggesting that future studies will need to identify possible dose-specific effects on attention functions.

### Limitations.

While our study already tested multiple markers of cognitive flexibility and attention, it was not yet incorporating tests of other domains that M_1_ mAChR modulating ligands might affect and which are compromised in psychiatric patient populations such as long-term memory and motivation (13, 81–83). Further tasks, where we can extract measures for these domains would be important additions for a more comprehensive characterization of possible M_1_ mAChR dependent behaviors (*SI Appendix*, *Supplemental Discussion*). Such an expansion of extracted measures would align well with efforts to develop multi-task batteries for NHPs covering a wide range of cognitive domains (53, 84–87).

### Conclusion.

In summary, the M_1_ PAM VU0453595 produced selective improvements in cognitive flexibility in the absence of adverse side effects. The results were obtained with cognitive tasks that tap into real-world cognitive demands for adjusting to the changing relevance of visual objects. This result pattern suggests that M_1_ PAMs will be powerful targets for drug discovery efforts to augment cognitive flexibility.

## Methods and Materials

### Subjects.

Four adult male rhesus macaques (*Macaca mulatta*) were separately given access to a touchscreen Kiosk Station attached to their housing unit where they performed a visual search attention task and a feature-reward learning task (85) (Fig. 1*A*) (*SI Appendix*, *Supplemental Materials*).

### Compounds and Procedures.

The scale up of the M_1_ PAM VU0453595 used in the present study was synthesized at the Molecular Design and Synthesis Center within the Vanderbilt Institute of Chemical Biology, Vanderbilt University, School of Medicine (30, 33) and mixed with a vehicle of 18 g of strawberry yogurt and 2 g of honey provided to the monkeys in a small paper cup (oral administration). All monkeys received vehicle or VU0453595 2 h prior to the start of behavioral performance and were observed to ensure full consumption of vehicle or VU0453595. VU0453595 was administered once per week to allow appropriate washout. Based on the weight of each animal, its volume was calculated for 0.3, 1, and 3 mg/kg doses. Side effects were assessed 15 min following VU0453595 administration and after completion of the behavioral performance with a modified Irwin Scale for rating autonomic nervous system functioning (e.g., salivation) and somatomotor system functioning (e.g., posture and unrest) (43, 88–90). Furthermore, monkeys’ behavioral status was video-monitored throughout task performance.

The pK for VU0453595 has previously been reported in cynomolgus macaques where peak concentrations at 3 mg/kg dosing occurred ~2 h after oral administration (80). The same study found changes to qEEG spectral power 0 to 4 h after VU0453595 administration in macaques, consistent with the time window for behavioral performance in our study. While changes in qEEG spectral power were also observed in mice, M_1_-KO mice did not exhibit these changes. Furthermore, the agonist and PAM activity of VU0453595, through calcium mobilization assays have been previously reported in M_1_-expressing CHO cells (22).

### Behavioral Paradigms.

Monkeys performed a sequence of two tasks in a single behavioral session including an initial VS task block, 21 reward learning task blocks and finally, a second visual task block. Rewarded and unrewarded objects in the VS task and FRL task were multidimensional, 3D rendered objects (named “Quaddles”) (91) that shared a variable number of different feature dimensions (colors, shapes, arms and/or body patterns). The VS task varied the perceptual target-distractor similarity by changing the average number of common features between distractors and the target object. The FRL task varied the complexity of the feature space by varying features of objects in only one or two feature dimensions from trial to trial.

At the start of each session, animals performed a VS task block and again at the end of each session, they performed a second VS task block. Each VS block contained an initial ten “familiarization trials” followed by 100 search trials. During the familiarization trials, only the rewarded object was presented on screen, without any distracting objects. The rewarded object was made up of three features of three different feature dimensions. The 10 familiarization trials were followed by a set of 100 search trials, where the rewarded object (learned during the familiarization trials) was always presented amongst 3, 6, 9, or 12 (counter-balanced and randomly selected) distracting objects (Fig. 1*B*, *Bottom*). These distracting objects could each share 0, 1, or 2 of the three features with the target. Animals received fluid reward for touching the learned target object presented during that block’s familiarization trials. Each of the two VS blocks in a daily session was accompanied by one of five patterned background images, selected without replacement daily. These images bore no relationship to the target objects and served to cue the animal to the task rule in contrast to the FRL task which contained a neutral gray background.

Between the two VS blocks, the animals performed 21 blocks of the FRL task where they had to identify the single feature value associated with high reward probability (85%). In each block (35 to 60 trials) of the FRL task, animals were required to learn, by trial-and-error, which single feature was associated with the reward. The FRL task indexes cognitive flexibility by testing how fast subjects learn which feature is rewarded when the feature-reward rule switched between blocks. The newly rewarded feature after the uncued block switches could be from the same or from a different feature dimension than the previously rewarded feature. This makes the task similar to attentional set-shifting tasks, but different by using a larger set of features that varied within and across sessions in order to control task difficulty. In each trial, three objects were shown that varied either in the features of one feature dimension (e.g., each object having different colors or body shapes), or that varied in features of two feature dimensions (e.g., each object having different colors and body shapes). Thus, in a single trial, no two objects contained any overlap in the presented features (Fig. 1*B*, *Top*). Choosing the object with the correct feature was rewarded with a probability of 0.85. Blocks where only 1 feature dimension varied (low distractor load) were easier as there were less distracting features than in blocks with two varying feature dimensions (high distractor load). Blocks switched after the completion of a minimum of 35, 40, or 45 trials (random jittering) if a performance threshold of ≥ 80% in the previous 10 trials was reached. Otherwise, blocks would switch after the completion of 60 trials.

### Statistical Analysis.

Data were analyzed with standard nonparametric and parametric tests with test statistics, *P* values, and effect sizes reported where appropriate in text. For detailed statistical methods, please see the *SI Appendix*.

### Financial Disclosures.

The authors declare no competing financial interests.

## Supplementary Material

Appendix 01 (PDF)

## Data Availability

.mat and .m files data have been deposited in Github (https://github.com/att-circ-contrl/PNAS_VU595_code-data) (92).
