# Supplementary material for: M1-selective muscarinic allosteric modulation enhances cognitive flexibility and effective salience in nonhuman primates
Source: Proc Natl Acad Sci U S A. 2023 Apr 27;120(18):e2216792120. doi: 10.1073/pnas.2216792120 (PMC10161096; doi:10.1073/pnas.2216792120)
Supplement: Supplementary file 1 — Appendix 01 (PDF) [file pnas.2216792120.sapp.pdf]

Supplemental Information for

M_1 selective muscarinic allosteric modulation enhances cognitive flexibility and effective salience in nonhuman primates

Seyed A. Hassani[1], Adam Neumann[1], Jason Russell[2,3], Carrie K. Jones[2,3], Thilo Womelsdorf[1,4]

[1]*Department of Psychology, Vanderbilt University, Nashville, TN 37240.*
[2]*Department of Pharmacology, Vanderbilt University, Nashville, TN 37240.*
[3]*Warren Center for Neuroscience Drug Discovery, Vanderbilt University, Nashville, TN 37240.*
[4]*Department of Biomedical Engineering, Vanderbilt University, Nashville, TN 37240.*

Content:

Supplemental Materials and Methods

Supplemental Results

Supplemental Discussion

Supplemental Figures

Supplemental Tables

Supplemental Materials and Methods

Subjects

All animal related experimental procedures were in accordance with the National Institutes of Health Guide for the Care and Use of Laboratory Animals, the Society for Neuroscience Guidelines and Policies, and approved by Vanderbilt University Institutional Animal Care and Use Committee.

Four pair-housed adult male rhesus macaques (*Macaca mulatta*), 7-11 years old and weighing ~8-15 kg were subjects in this experiment. Monkeys in each pair were separately given access to a cage-mounted Kiosk Station attached to their housing unit uniformly at either 11am (monkeys Ig and Ba) or at 1pm (monkeys Re and Si). Each monkey was overtrained and engaged with and completed a visual search attention task and a flexible feature-reward learning task via a touchscreen interface (1) with the software being controlled by the Unified Suite for Experiments (USE) (2).

Of the four monkeys, two (monkeys Si and Ig) had previously been involved in a similar study utilizing the acetylcholinesterase inhibitor donepezil (3) with over 6 months between experiments for washout. Prior to the donepezil experiments, monkey Ig had also previously been exposed to a

different experimental M_1 PAM. Monkeys Ba and Re were naïve to VU0453595 and other neurological or psychiatric medications.

Comparison table

The comparison table (Table 1) between the best dose of donepezil and VU0453595 was based on the data collected in a previous study (3) but for all measures, identical methods were applied to both datasets consistent to what is described here.

Statistics

Within the feature-reward learning (FRL) task, we use trials-to-criterion to quantify learning efficiency with the criterion being defined as the first trial after at least 1 error which preceded a string of 10 trials with 70% or greater performance. Note that 70% trials-to-criterion measure is different from the backward-looking threshold of 80% which determined the switching of blocks during FRL task performance. The 70% performance threshold is different from our previous work (3) which was set to 80% performance, which was and still is the criterion for block switches in the FLR task. We found this new threshold to better reflect the occurrence of learning and led to only a mean 0.37 (0 median) trial difference in baseline trials-to-criterion overall. The comparisons between VU0453595 and donepezil were made using the same definition for each measure.

Block switches in the FRL task were labelled based on the status of the target feature relative to the previous block as extra-dimensional, intra-dimensional or as involving a novel target feature dimension. For novel target blocks, the rewarded feature dimension was not present in the previous block, independent of the present or previous block's dimensionality. Similarly, intra-dimensional shift blocks involved the same rewarded feature dimension but a different rewarded feature (e.g. a different color) as the previous block, independent of their dimensionality. However, for extra-dimensional shift blocks, the previous block must have been a high load (objects varying in 2 feature dimensions) block where the current block's target feature was from the previous one's distracting feature dimension. Extra-dimensional shift blocks themselves could be either low or high distractor load.

In the FRL task, for each session, reaction times (RTs) were averaged and smoothed using a 5 trial shifting window for low and high distractor load blocks separately (**Fig. 2A,B**). We then defined the time to plateau as the first trial per session, excluding trial two, where the RTs began to decrease.

Perseverative errors were quantified based on the features of the erroneously chosen object. The consecutive errors could be made with objects containing the same feature from the distracting or target feature dimensions. The proportion of perseverative errors are reported as a percentage of all errors (**Fig. 2G,H**).

In order to account for temporally specific effects on learning efficiency with VU0453595, as seen with other cholinergic compounds (3), we applied a linear mixed effects model (LMEM) to the trials-to-criterion. The LMEM had three main effects: experimental condition, distractor load and temporal bin (thirds), while individual monkeys were treated as random effects:

$$Trial\ to\ criterion = ExpCond \times DistractorLoad \times TemporalThirds + (1|Monkey) + b + \varepsilon$$

Given the results of the LMEM and the maximal effect size with the first third of blocks in the FRL (**Fig. S1A**), all analyses for the FRL task used only the first third of blocks to capture the period where VU0453595 had its strongest effect on performance.

Effect sizes were reported as either eta squared values when referring to ANOVA results or Cohen's d when appropriate (i.e. when post-hoc analysis showed a significant effect at a single dose). The Cohen's d was computed by directly comparing vehicle to the significant dose using this formula:

$$d = \frac{M_2 - M_1}{\sqrt{\frac{(n_1 - 1)s_1^2 + (n_2 - 1)s_2^2}{n_1 + n_2 - 2}}}$$

Supplemental Results

Feature-reward learning task

After reaching performance criterion, VU0453595 also resulted in higher plateau accuracy (compound condition main effect: (F(3,1672) = 3.22, p = .02; η^2 = .005); low distractor load accuracy: 90.9% (SE: 1.9%); 95.9% (SE: 0.9%); 90.9% (SE: 1.8%); 91.0% (SE: 0.7%) for 0.3, 1, 3 mg/kg and vehicle respectively; high distractor load accuracy: 74.4% (SE: 3.0%); 80.5% (SE: 2.9%); 84.8% (SE: 2.3%); 77.9% (SE: 1.1%) for 0.3. 1, 3 mg/kg and vehicle respectively) (**Fig. S1B**). The middle dose of VU0453595 (1 mg/kg) also increased the proportion of blocks in which animals reached the learning criterion of 70% over the subsequent 10 trials using a forward looking 10 trial window (F(3,1369) = 2.93, p = .03; η^2 = .006; Tukey's, p = .02). Animals reached the learning criterion of 70% accuracy over 10 successive trials in 90.5% (SE: 2.4; low load) and 72.1% (SE: 3.7%; high load) of blocks in the vehicle condition. Tukey's HSD multiple comparisons test among proportions revealed that at the 1 mg/kg dose, VU0453595 significantly increased the proportion of learned blocks in the low load condition to 98.7% (SE: 2.6%) (p = .04) (**Fig. S1C**).

Visual search task

In the first VS block, target detection times across distractor conditions were not different with VU0453595 relative to vehicle control (F(3,944) = 1.67, n.s.; η^2 = .004), with the exception of faster target detection times in the second VS block at the 3 mg/kg dose (experimental condition main effect: F(3,944) = 3.67, p = .01; η^2 = .008; Tukey's, p < .05). With regards to performance, in the VS block at the end of the session there were no significant effects, while in the first VS block there was a significant main effect of compound (F(3,944) = 3.80, p = .01; η^2 = .010) with reduced accuracy at 3 mg/kg dose (Tukey's, p = .04) irrespective of the number of distractors.

Despite the lack of set size effects, the raw target detection times were overall significantly faster with the 1 mg/kg dose in the second block (F(3,708) = 4.67, p = .003; η^2 = .018; Tukey's, p = .02) with more improvement with high target-distractor similarity (cohen's d = -.447) than low target-

distractor similarity (cohen's d = -.427) (**Fig. 3E**). There was also a general reduction in performance during the first block ($F(3,708) = 2.84$, $p = 0.04$; $\eta^2 = 0.011$) (**Fig. 3F**). We also tested if there was a speed-accuracy trade-off at any dose of VU0453595 during either the first or second VS block. We found no significant changes in the speed-accuracy relationship between vehicle and any of the administered doses of VU0453595 (**Figure S2**).

Characterization of adverse cholinergic side effects

Each subject was observed during their consumption of VU0453595 stirred into a strawberry yogurt and honey vehicle (20 g total) placed in a small paper cup. Of the 4 subjects, 1 placed the entire cup immediately in their mouth while the other 3 consumed all of the paper cup's content before either ripping it and licking it clean or eating parts of the paper cup alongside the yogurt. None of the subjects had any day where they spilled an observable volume of yogurt. A modified Irwin test, which measures cholinergic effects on the autonomic and somatomotor systems, was applied to all subjects twice daily (**Table S1**). Subjects were observed throughout the experiment through a video monitoring system (**Figure 1A**) and formally evaluated for the modified Irwin test once, ~110 minutes after administration (immediately before start of task), and a second time, immediately after subjects finished all behavioral tasks for their session (< 2 hours after the first assessment). Ratings of 0, 1 or 2 were assigned to each item on the test reflecting no change, a slight change, or a significant change respectively. During the first 1 mg/kg dose of VU0453595, 4/4 monkeys experienced slight changes in arousal while 2/4 monkeys experienced slight unrest and 1/4 experienced a slight increase in yawning. No other symptoms were observed beyond the first 1 mg/kg dosing. At the 3 mg/kg dose, 4/4 monkeys experienced slight unrest, 3/4 monkeys experienced slight changes in arousal and a single monkey experienced vasodilation (redness of the face). Most of the symptoms observed at the 3 mg/kg dose also occurred during the first 3 mg/kg dosing event. Only a single monkey had any symptoms post-task completion, once at the 1 mg/kg dose and once at the 3 mg/kg dose, both instances involved a slight change in arousal. No changes were observed at the 0.3 mg/kg dose.

Supplemental Discussion

M_1 PAM literature review

We compiled an exhaustive summary of M_1 PAM NHP (4–9) and rodent papers (4, 7, 10–35) to the best of our knowledge (**Table S2 & S3** respectively) and identified the tasks they utilized and which cognitive domains their extracted measures were informative of. A large majority of all of the identified papers contain some pharmacological challenge (i.e. scopolamine, amphetamine, PCP, haloperidol etc) in order to demonstrate the efficacy of the candidate M_1 PAM (5/6 NHP papers and 16/28 rodent papers; of the remaining rodent papers 7 involved a genetic disease model and another 4 contained some lesion or prion component). In contrast, 0/6 NHP studies and 4/28 rodent studies used varying task parameters in order to manipulate cognitive demand for pharmacological testing. One potential reason for this is the relatively simple, and easy-to-train, design of the commonly used behavioral tasks, where over trained animals have near ceiling performance. Such tasks also suffer from being only informative of a single cognitive domain and require modification for further utility. For example, the Morris water maze was used in 8 rodent M_1 PAM studies to assay learning and memory, however, the addition of a reversal component allowed for measures of cognitive flexibility and a dissociation of the impact of VU0486846 in

one study (36; **see Table S3**). Both **Tables S2** (37–48) and **S3** (41, 49–51) also include a select few non-M_1 PAM cholinergic drug studies. Although focusing on the symptoms of diseases of interest is an attractive approach for identifying the efficacy of a candidate compound, it is important to dissociate its impact on multiple cognitive domains in order to identify differences in optimal dosing and potential cognitive trade-offs (e.g. 3, 19).

Possible M_1 agonism

Although *in vitro* data suggests little to no agonistic properties of VU0453595 (21), we cannot completely rule out the possibility that the inverted-U shaped responses observed with this compound may be due, in part, to ago-PAM activity at the highest dose tested *in vivo*. This would suggest that the endogenous signaling at the M_1 mAChR supporting cognitive flexibility is sensitive to exogenous intervention. The possible agonism of M_1 PAMs such as VU0453595 *in vivo* will be the subject of future studies.

Possible contributions of M_1 potentiation of memory or motivation/effort control

The current study dissociated the relative importance of an M_1 PAM (VU0453595) for cognitive flexibility and attentional filtering and contrasted these effects to those of donepezil (**Table 1**). The functional dissociation of the effects highlights the importance of a multi-task paradigm for understanding ligand actions on behavior (3, 45, 52) and supports efforts to develop multi-task batteries covering a wide range of cognitive domains (1, 45, 52–54). While our study tested already multiple markers of cognitive flexibility and attention, it was not yet incorporating tests of domains that M_1 modulating compounds might also affect. For example, scopolamine challenges have long suggested that M_1 mAChRs in the medial temporal lobe support longer-term memory processes (55–57), making it possible that M_1 mAChR modulation might have positive consequences in this domain.

Motivation and the ability to control effort are other domains that we did not test and which some studies have suggested to be modulated by mAChRs. The task we deployed varied cognitive load which inevitably increases difficulty and the amount of effort subjects needed to exert. Although we did not control for motivational factors explicitly, visual inspection suggested it was not modulated by VU0453595 because the learning improvements were somewhat more pronounced at lower than higher load in the learning task and did not vary with increasing distractor difficulty (target-distractor similarity) in the search task. These findings resonate with the results of a scopolamine challenge study in NHP that found no effects of increasing difficulty in a stimulus-location association learning task (58). However, when testing for a memory load effect with a visuo-spatial paired associate task, Taffe and colleagues (59) found that scopolamine reduced performance particularly when 3 or 4 stimulus-object associations needed to be learned and retrieved but not when 1 or 2 associations were involved. Such a memory load differs from the cognitive load that we imposed by increasing the number of distracting features in the learning task and from the perceptual load that we varied with increasing target distractor similarity. However, it will be important to identify in future studies which motivation or load dependent processes are modulated specifically by M_1 selective mAChR modulation.

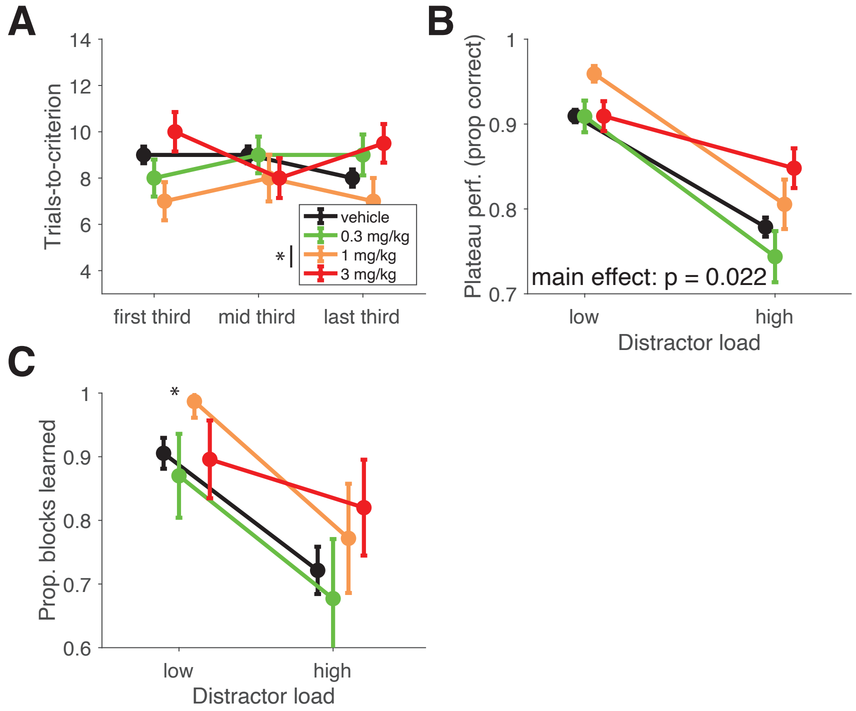

Figure S1. VU0453595 enhances multiple measures of learning performance (**A**) The median trials-to-criterion, visually combined for low and high distractor load conditions temporally split by their presentation within a session (7 blocks in each third) for vehicle, 0.3, 1 and 3 mg/kg doses of VU0453595. The LMEM used the experimental condition, temporal bin (thirds) and distractor load as fixed effects. There was significantly faster learning with 1 mg/kg which showed the strongest effect size during the first third of FRL blocks (1 mg/kg fixed effect: t(3674) = -2.67, p = .008; first third Cohen's d = -.228; overall Cohen's d = -.061). (**B**) Average performance in the final 10 trials of low and high distractor load blocks of the FRL task. For the low distractor load blocks, plateau performance was 90.95% (SE: 0.73), 90.90% (SE: 1.86), 95.92% (SE: 0.92) and 90.94% (SE: 1.76) for vehicle, 0.3, 1 and 3 mg/kg doses of VU0453595 respectively. For the high distractor load blocks, plateau performance was 77.86% (SE: 1.12), 74.37% (SE: 3.01), 80.54% (SE: 2.91) and 84.80% (SE: 2.34) for vehicle, 0.3, 1 and 3 mg/kg doses of VU0453595 respectively. There was a significant main effect of experimental condition (F(3,1672) = 3.22, p = .022; η^2 = .005) but post hoc analysis (Tukey's) showed no single dose as significantly different from vehicle. (**C**) Average proportion of learned blocks (defined as blocks that reached the 70% performance over 10 trials; the same measure as trials-to-criterion) per session in the FRL task. For the low distractor load blocks, the proportion of blocks learned was 90.54% (SE: 2.42), 87.00% (SE: 6.59), 98.68% (SE: 2.56) and 89.58% (SE: 6.11) for vehicle, 0.3, 1 and 3 mg/kg doses of VU0453595 respectively. For the high distractor load blocks, the proportion of blocks learned was 72.14% (SE: 3.71), 67.71% (SE: 9.35), 77.17% (SE: 8.58) and 82.00% (SE: 7.53) for vehicle, 0.3, 1 and 3 mg/kg doses of VU0453595 respectively. Pair-wise comparisons between the VU0453595 doses and vehicle revealed a significant improvement at the low distractor load with the 1 mg/kg dose (Tukey's multiple comparison test among proportions: q = 4.082, q_{crit} = 3.633) and no significant changes at the high distractor load (Tukey's multiple comparison test among proportions, n.s.).

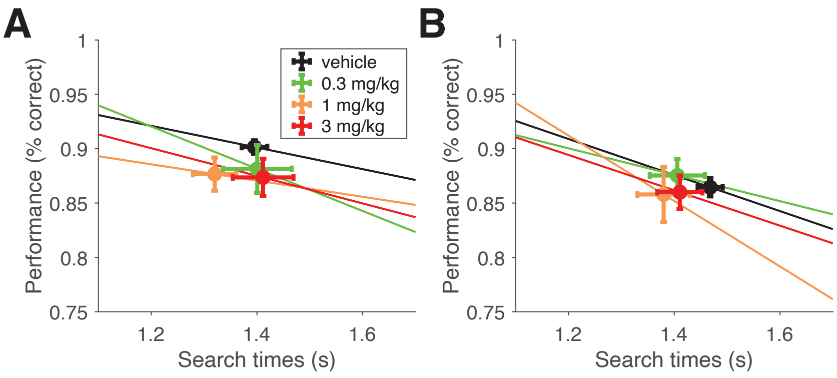

Figure S2. VU0453595 does not change the speed-accuracy trade-off in the visual search task. (**A**) The mean search times and block performance across all of the first VS blocks. The lines represent the linear relationship between the search times and performance for these blocks. No significant difference in the relationship between performance and search times was observed (all n.s.; fisher r to z transformation). (**B**) Same as A but for the second VS block. No significant differences were observed in the relationship between performance and search times (all n.s.; fisher r to z transformation).

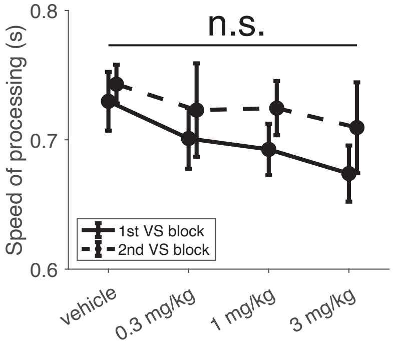

Figure S3. VU0453595 does not impact the speed of processing. The speed of processing for the first and second VS blocks, defined as the time animals took to touch the only object on screen (during familiarization trials). In the first VS block, speed of processing was 0.730 s (SE: 0.023), 0.701 s (SE: 0.024), 0.693 s (SE: 0.020) and 0.674 s (SE: 0.022) for vehicle, 0.3, 1 and 3 mg/kg doses of VU0453595 respectively. In the second VS block, speed of processing was 0.743 s (SE: 0.015), 0.723 s (SE: 0.036), 0.725 s (SE: 0.021) and 0.710 s (SE: 0.035) for vehicle, 0.3, 1 and 3 mg/kg doses of VU0453595 respectively. No significant changes were observed for the first ($F(3,236) = .56$, n.s.) or second VS blocks ($F(3,236) = .35$, n.s.).

	VU0453595	0.3 mg/kg		1 mg/kg		3 mg/kg	
	Observation	Pre-task	Post-task	Pre-task	Post-task	Pre-task	Post-task
Autonomic Nervous System	Salivation	-	-	-	-	-	-
	Lacrimation	-	-	-	-	-	-
	Urination	-	-	-	-	-	-
	Defecation (amount)	-	-	-	-	-	-
	Defecation (consistency)	-	-	-	-	-	-
	Emesis	-	-	-	-	-	-
	Miosis	-	-	-	-	-	-
	Mydriasis	-	-	-	-	-	-
	Ptosis	-	-	-	-	-	-
	Exophtalmos	-	-	-	-	-	-
	Piloerection	-	-	-	-	-	-
	Respiratory Rate	-	-	-	-	-	-
	Yawn	-	-	+	-	-	-
	Vasodilation	-	-	-	-	+	-
	Vasoconstriction	-	-	-	-	-	-
	Irritability	-	-	-	-	-	-
	Body Temp.	-	-	-	-	-	-
Somatomotor Systems	Physical Appearance	-	-	-	-	-	-
	Tremor	-	-	-	-	-	-
	Leg Weakness	-	-	-	-	-	-
	Catalepsy	-	-	-	-	-	-
	Visuo-Motor Coordination	-	-	-	-	-	-
	Posture	-	-	-	-	-	-
	Unrest	-	-	+	-	+	-
	Stereotypies	-	-	-	-	-	-
	Arousal	-	-	+	+	+	+
	Sedation	-	-	-	-	-	-
	Oral Dyskinesia	-	-	-	-	-	-
	Bradykinesia	-	-	-	-	-	-
	Dystonia	-	-	-	-	-	-

Table S1. A summary of observed dose-limiting side effects. The effect of VU0453595 (0.3, 1 and 3 mg/kg PO) on autonomic and somatomotor system function were evaluated. The mean score of 4 monkeys was classified as follows: - no effect; + 0-0.15; ++ 0.16-0.3; +++ 0.31-0.45

Table S2. Summary of NHP studies with compounds acting at the M_1 receptor. Light gray: sample studies without an M_1 PAM; white: studies using M_1 PAMs; dark gray: summary. Background color on tasks defines their cognitive domain they pertain to. White: no task; green: working memory; blue: learning and memory; purple: executive functioning; orange: attention and/or vigilance; cyan: social; yellow: motor control or locomotion; red: motivation.

Reference	Sample/Subject Info	Ligands & Doses	Reported Adverse Effects	Task(s)	Challenge	Number of Determinations	Cognitive Domain(s)	Positive Results
Glick & Jarvik 1970	8 rhesus macaques	Muscarinic antagonist (Scopolamine): 0.025, 0.05, 0.1 mg/kg (im)	None reported	1. Delayed match to sample task	Behavioral (delay)	At least 2 per dose	Working memory	All doses reduced accuracy and response frequency
Bartus & Johnson 1976	8 rhesus macaques (m; adolescent)	Muscarinic antagonist (Scopolamine): 0.03, 0.04, 0.05 mg/kg (im)	Ptosis, pupil dilation	1. Delayed match to sample task	Behavioral (delay)	1 per dose	Working memory	Severe disruption of performance
Buccafusco et al., 2003	12 rhesus macaques (m/f; >20 y.o)	AChE-I (Donepezil): 0.01, 0.025, 0.05, 0.1 mg/kg (im)	None reported	1. Delayed match to sample	Behavioral (delay)	1 per dose	Working memory	Enhanced accuracy @ 0.025mg/kg (m) and @ 0.1mg/kg (f)
Buccafusco & Terry 2004	17 rhesus macaques (m/f; 9-29 y.o.)	AChE-I (Donepezil): 10, 25, 50, 100 ug/kg (im)	None reported	1. Delayed match to sample	Behavioral (delay)	1 per dose	Working memory	Enhanced accuracy during medium & long delays @ 25ug/kg
Buccafusco et al., 2008	32 rhesus macaques (m; avg. 18.8 y.o.)	AChE-I (Donepezil): 5, 10, 25, 50, 100, 125 ug/kg (im & po)	None reported	1. Delayed match to sample	Scop.	1, 4, 4, 2, 1, 1 per dose respectively	Working memory	Partial rescue @ 50ug/kg (po)
Rupniak et al., 1997	9 rhesus macaques (m; young adult)	AChE-I (Donepezil): 0.003, 0.01, 0.03, 0.04, 0.05, 0.06, 0.1, 0.5, 1, 1.75 mg/kg (im)	Tremors, jerking, retching, mouth movements, salivation, pallor & lethargy @ 2 mg/kg	1. Spatial delayed response task	Scop.	1 per dose	Working memory	Partial rescue @ 0.5, 1 & 1.75 mg/kg
				2. Visual recognition task	None	1 per dose	Attention/vigilance	Pre-treatment enhanced performance @ 0.03 (best) & 0.05 mg/kg
Oliveira et al., 2021	25 (5 per treatment group) black tufted-ear	AChE-I (Donepezil): 0.5 mg/kg (sc)	None reported	1. Novel object recognition task	A: Scop.<br>B: MK-801 challenge	1 per dose	Learning and memory	A: rescue of novel object exploration preference.<br>B: no effect

	marmosets (f/m; 4.5-8 y.o.)				(NMDA antagonist)			
Tsukada et al., 2004	10 rhesus macaques (m; half ~5 & half ~20 y.o.)	AChE-I (Donepezil): 50, 250 ug/kg (iv)	None reported	1. Oculomotor delayed response task	Monkey age	1 per dose	Working memory	Partial rescue @ both 50 and 250 ug/kg in old monkeys. Trend in young monkeys (n.s.)
				2. Visually guided saccade task	Monkey age	1 per dose	Motor control; attention/vigilance	No effect
Taffe et al., 1999	6 rhesus macaques (m; ~4 y.o.)	Muscarinic antagonist (Scopolamine): 3, 10, 14, 17, 24 ug/kg (im)	None reported	1. Delayed non-match to sample	Behavior (delay)	Tasks 1-5: 1 per dose for 4 separate doses *all animals were dosed at 3, 10 & 17 ug/kg. 3 of the animals also received 24 ug/kg dose while the other 3 received a 14 ug/kg dose	Working memory	Reduced accuracy (no delay interval interaction)
				2. Self-ordered spatial task	Behavior (number of objects)		Working memory; attention/vigilance	Reduced accuracy (with interaction)
				3. Reaction time	RT		Speed of processing; attention/vigilance	Increased movement time only @ 14 &17ug/kg
				4. Progressive ratio task	Behavior (satiation)		Motivation	Reduced reinforcer acquired @ 14, 17, 24ug/kg
				5. Bimanual motor task	RT		Motor control	Increased latency @ 14, 17 & 24ug/kg
Knakker et al., 2021	6 rhesus macaques (m; ~5 y.o.)	AChE-I (Donepezil): 100, 200 ug/kg (im)	None reported	1. Delayed match to sample task	Scop.	1 per dose	Working memory	Partial reversal during medium delay @ 200 ug/kg
Callahan 1999	6 rhesus macaques (m/f; 12+ y.o.)	AChE-I (Tacrine): 0.03, 0.1, 0.32, 1 mg/kg (im) Muscarinic agonist (Milameline): 0.001, 0.003, 0.01 mg/kg (im)	None reported	1. Continuous performance task	Scop.	1 per dose	Attention/vigilance	Partial to full rescue of response omissions @ 0.32 mg/kg Tacrine. Partial rescue of response omissions @ 0.003 or 0.01 mg/kg milameline. Partial rescue @ tested combinations with stronger effect than the same dose of either ligand alone
Callahan et al., 2013	7 rhesus macaques (m/f; aged)	AChE-I (Donepezil): 0.003, 0.01, 0.025, 0.05, 0.1, 0.2 mg/kg (po)	None reported	1. Delayed match to sample task	Behavioral (delay)	6-7 dosing events total	Working memory	Accuracy enhancement with donepezil @ 0.01, 0.025, 0.05, 0.1 & 0.2 mg/kg. Accuracy enhancement with donepezil (0.003 mg/kg) + PNU-120596 @ 3 & 10 mg/kg (PNU-120596 alone did not show result in significant improvement)

		Nicotinic (α7) PAM (PNU-120596): 1, 3, 10 mg/kg (po)						
Gould et al., 2020	8 cynomolgus macaques (m; 4-8 y.o.)	M_1 PAM (VU0453595): 3, 10, 30 mg/kg (ig) M_1/M_4 agonist (Xanomeline): 1, 3 mg/kg (sc) AChE-I (Donepezil): 3, 10 mg/kg (po)	Increased urination, reduced respiration, changes in posture, motor coordination, leg weakness @ 30 mg/kg VU0453595	None (qEEG study)	N/A	1 per dose	N/A	VU0453595: Beta power (18-30Hz) increased @ 30mg/kg, gamma power (30-50Hz) increased @ 10 & 30mg/kg
Kurimoto et al., 2019	4 (per group) cynomolgus macaques (m; 3-5 y.o.)	M_1 PAM (TAK-071): 0.3, 1, 3 mg/kg (po) AChE-I (Donepezil): 0.3, 3 mg/kg (po) M_1/M_4 agonist (Xanomeline): 1 mg/kg (sc)	None reported	None (qEEG study)	Scop. (increased delta, theta and alpha power)	1 per dose	N/A	TAK-071: alone it decreased alpha power @ 3 mg/kg and theta + alpha power @ 1 mg/kg. It lead to partial rescue of alpha power @ 1 mg/kg (delta lowered but n.s.). It also lead to partial rescue of alpha and delta power @ 3 mg/kg (delta lowered but n.s.) Donepezil: alone it increased alpha power @ 0.3 mg/kg. It also lead to partial rescue of delta, theta and alpha power. Xanomeline: partial rescue of delta, theta and alpha power (all trends; n.s.).
Uslaner et al., 2013	6 rhesus macaques (m; 4-6 y.o.) 6 cynomolgus macaques (f; ~15 y.o.)	AChE-I (Donepezil): 0.3 mg/kg (po) M_1 PAM (PQCA): 3, 10, 30 mg/kg (po)	None reported	1. Object retrieval detour task	Scop.	1 per dose	Reasoning & problem solving (exec. functioning)	Partial rescue @ 10 & 30mg/kg PQCA
				2. Self-ordered spatial task	Scop.	1 per dose	Working memory	Partial rescue @ 10mg/kg PQCA & 0.3mg/kg donepezil
Uslaner et al., 2018	8 rhesus macaques (m; adult)	M_1 PAM (MK-7622): 0.1, 0.3, 1 mg/kg (po)	None reported	1. Object retrieval detour task	Scop.	1 per dose	Reasoning & problem solving (exec. functioning)	Partial rescue @ 0.3 & 1 mg/kg
Lange et al., 2015	18 rhesus macaques (m; adult)	M_1 PAM (PQCA): 0.3, 1, 3, 10, 30 mg/kg AChE-I (Donepezil): 0.1, 0.25, 0.3, 1, 3 mg/kg	None reported	1. Paired-associates learning task	Scop.	1 per dose (po)	Working memory	Partial rescue @ 10 & 30 mg /kg PQCA (po)
				2. Continuous performance task	Scop.	1 per dose (im)	Attention/vigilance & impulsivity (exec. functioning)	Partial rescue @ 0.3 & 1 mg/kg PQCA (im)

Vardigan et al., 2015	8-12 rhesus macaques (m/f)	M_1 PAM (PQCA): 3, 30, 50 mg/kg (po)<br>M_1/M_4 agonist (Xanomeline): 0.03, 0.1, 0.3 mg/kg (im)<br>AChE-I (Donepezil): 0.3, 0.56, 1, 1.8, 3, 5 mg/kg (po)	Increase in feces (weight) @ 5 mg/kg donepezil & 0.3 mg/kg xanomeline. Increase severity of salivation & emesis @ 0.3 mg/kg xanomeline	1. Object retrieval detour task	Scop.	1 per dose	Reasoning & problem solving (exec. functioning)	Partial rescue @ 1, 1,.8, 3 mg/kg donepezil & 0.1 mg/kg xanomeline; partial rescue also @ 0.3 mg/kg donepezil + 3 mg/kg PQCA
Current study	4 rhesus macaques (m; 7-11 y.o.)	M_1 PAM (VU0453595): 0.03, 0.1, 0.3 mg/kg (po)	None reported	1. Feature-reward learning task	Distractor load	7 per dose	Learning and memory; cognitive flexibility (exec. functioning)	Better performance, faster learning and less perseverations @ 0.1 mg/kg
				2. Visual search	Distractor interference & perceptual interference	7 per dose	Speed of processing; attention/vigilance; working memory	No reliable change
Total studies utilizing PAMs: 6	4-18 monkeys with a median of 8	M_1 PAMs:<br>-VU0453595 (1/6)<br>-TAK-071 (1/6)<br>-MK-7622 (1/6)<br>-PQCA (3/6)	Reported for PAMs (VU0453595) and other cholinergic agents (donepezil, xanomeline)	2/6 tasks had no behavioral component<br>2/6 had 2 tasks<br>2/6 had only 1 task	Studies with behavior and even 1 of the 2 studies without behavior used a scopolamine challenge.	Average number of determinations: 1 (6/6 had 1 determination per dose)	Cognitive domains tested:<br>- Reasoning & problem solving (3/4)<br>-Working memory (2/4)<br>-Attention/ Vigilance (2/4)	Rescue of scopolamine challenged behavior in all cases with least 1 dose of the tested PAM.

Table S3. Summary of rodent studies with compounds acting at the M_1 receptor. Light gray: sample studies without an M_1 PAM; white: studies using M_1 PAMs; dark gray: summary. Background color on tasks defines their cognitive domain they pertain to. White: no task; green: working memory; blue: learning and memory; purple: executive functioning; orange: attention and/or vigilance; cyan: social; yellow: motor control or locomotion; red: motivation/drug abuse.

Reference	Sample/Subject Info	Ligands & Doses	Reported Adverse Effects	Task(s)	Challenge	Number of Determinations	Cognitive Domain(s)	Positive Results
Buccafusco et al., 2008	Albino Wistar and Long-Evans rats (m; 2-3 m.o.)	AChE-I (Donepezil): 1, 2 mg/kg (sc)	None reported	1. Morris water maze	Scop.	1 per dose	Learning and memory	Partial rescue @ 2mg/kg
				2. Delayed stimulus discrimination task	Scop.	1 per dose	Working memory	Partial rescue @ 1mg/kg
Lebois et al., 2017	5XFAD mice (6 m.o.)	M_1 agonist (VU0364572): 10 mg/kg (in drinking water)	None reported	1. Morris water maze	Mice with elevated beta amyloid	10 mg/kg daily dosing for 4 months (from 2-6 m.o.)	Learning and memory	Partial rescue after 4 months of chronic dosing
Digby et al., 2012	Sprague-Dawley rats (m)	M_1 allosteric agonist (VU0364572): 0.03, 0.056, 0.1, 0.3, 0.56 mg/kg (ip) M_1 allosteric agonist (VU0357017): 0.03, 0.1, 0.3, 0.56, 1, 3 mg/kg (ip)	None reported	1. Morris water maze	None	5 per dose	Learning and memory	Enhanced performance (swim distance) with VU0364572 @ 0.1 (best) & 10 mg/kg.
				2. Contextual fear conditioning	None	1 per dose	Learning and memory	Better acquisition of contextual fear with VU0364572 @ 0.056, 0.3 & 0.56 mg/kg as well as VU0357017 @ 0.1, 0.3, 0.56, 1 & 3 mg/kg
Xiong et al., 2019	C57BL/6 mice	M_1 agonist (77-LH-28-1): 5 ul of 5 uM via cannula (intra-cerebroventricular)	None reported	1. Morris water maze	GluA2 mutated mice	3 per dose	Learning and memory	Better (faster) reversal performance. No enhancement observed in GluA2 mutated mice.
Gould et al., 2020	Sprague-Dawley rats (4-6 m.o.) 10 C57BL/6NTac mice (m; 22-26 m.o.)	M_1 PAM (VU0453595): 3, 10, 30 mg/kg (ip) M_1 PAM (BQCA): 3, 10, 30 mg/kg (sc) AChE-I (Donepezil): 1, 3, 10 mg/kg (ip)	None reported	None (sleep/wake study)	N/A	N/A	N/A	BQCA increased duration awake and reduced REM and NREM sleep

	6 M_1 KO (m; 4-6 m.o.)							
Uslaner et al., 2013	Wistar Hannover rats (m)	AChE-I (Donepezil): 1.8 mg/kg (ip)<br>M_1 PAM (PQCA): 3, 10, 30 mg/kg (ip)	None reported	1. Novel object recognition	Scop.	1 per dose	Working memory	Partial rescue @ 10mg/kg PQCA & 3mg/kg donepezil
Chambon et al., 2011	Sprague-Dawley rats (m; adult)	M_1 PAM (BQCA): 5, 10 mg/kg (ip)	None reported	1. Novel object recognition task	Behavioral	1 per dose	Learning and memory	Retained familiar object memory @ 72h delay with BQCA 10 mg/kg (but not with vehicle or BQCA 5 mg/kg)
Chambon et al., 2012	Sprague-Dawley rats (m; adult)	M_1 PAM (BQCA): 10 mg/kg (ip)<br>M_1/M_3 agonist (Cevimeline): 1, 3 mg/kg (po)	Cevimeline increased salivation. None reported for BQCA.	1. Spontaneous alternation task	Scop.	1 per dose	Working memory	Partial rescue of alternations & trials completed @ 10 mg/kg BQCA
Bradley et al., 2017	Tg37 hemizygous mice	M_1 PAM (BQCA): 15, 20, 30 mg/kg (ip)<br>M_1 PAM (BQZ-12): 1.5, 5 mg/kg (ip)<br>AChE-I (Donepezil): 0.5, 1, 2.5 mg/kg (ip)<br>M_1/M_4 agonist (Xanomeline): 5, 10, 15, 30 mg/kg (ip)	None reported for BQCA (up to 30 mg/kg) or BQZ-12 (up to 5 mg/kg). Donepezil (1 & 2.5 mg/kg) & Xanomeline (10-30 mg/kg): piloerection, squinting, subdued and hunched posture. Donepezil also resulted in impaired mobility, laboured respiration, ataxia and paralysis.	1. Contextual fear conditioning	Prion disease	1 per dose	Learning and memory	Donepezil (0.5 mg/kg), Xanomeline (5 mg/kg) (controls), BQCA (15 mg/kg) & BQZ-12 (1.5 mg/kg) fully rescued behavior when administered before conditioning. Daily BQCA (15 mg/kg) treatment prolonged survival.
Dwomoh et al., 2022	Tg37 hemizygous mice	M_1 PAM (VU0486846): 10 mg/kg	None reported	1. Contextual fear conditioning	Prion disease	1 per dose	Learning and memory	VU0486846 (10 mg/kg) fully rescued behavior when administered before conditioning.

								Daily VU0486846 treatment resulted in slower disease progression and prolonged survival in some animals
Rook et al., 2018	Sprague-Dawley rats (m) *C57BL/6 mice (m; 7-8 w.o.) used for adverse effects testing	M_1 PAM (VU0486846): 1, 3, 10 mg/kg (ip)	Minor piloerection and pinna reflex loss @ 100 mg/kg (ip)	1. Novel object recognition	Behavior	1 per dose	Working memory	Enhanced recognition memory in rats @ 3 & 10 mg/kg
				2. Contextual fear conditioning	Risperidone challenge	1 per dose	Learning and memory	Rescue @ 10 mg/kg
Ma et al., 2009	12-16 (per group) B6SJL mice (m; 10 w.o.)	M_1 PAM (BQCA): 5, 10, 15, 20 mg/kg (ip)<br>M_1 allosteric agonist (TBPB): 10, 30 mg/kg (ip)<br>M_1 allosteric agonist (AC-42): 3, 10, 30 mg/kg (ip)	None reported	1. Contextual fear conditioning	Scop.	1 per dose	learning and memory	Full rescue @ 15 and 20 mg/kg
Puri et al., 2015	Tg2576 transgenic mice (f; 3-6 & 9-12 m.o.)	M_1 PAM (PQCA): 0.1, 1, 10 mg/kg (ip)<br>AChE-I (Donepezil): 0.1, 0.3, 1, 3 mg/kg (ip)	None reported	1. Novel object recognition task	Aged mice with elevated beta amyloid	1 per dose	Learning and memory	Recognition improved to comparable levels of WT aged mice @ 0.3, 1 mg/kg donepezil & 10 mg/kg PQCA.<br>Combining subthreshold doses (0.03 mg/kg donepezil & 1 mg/kg PQCA) also resulted in a similar enhancement.
Rook et al., 2017	C57BL/6 mice	M_1 PAM (VU6004256): 1, 3, 10 mg/kg (ip)<br>M_1 PAM (PF-06764427): 1, 3, 10 mg/kg (ip)<br>* Other M_1 PAMs were tested for adverse effects (BQCA; VU6004877; VU6006270; VU6006251; VU6005263; all ip)	Using 100 mg/kg of each ligand: BQCA, VU6004877, VU6006270, & PF-06764427 induced convulsions. VU6004256 induced mydriasis, piloerection, loss of some fine motor control.	1. Novel object recognition task	None (single condition)	1 per dose	Learning and memory	Recognition was enhanced with pretreatment of VU6004256 @ 3 & 10 mg/kg.<br>Trend for enhanced recognition (n.s.) with pretreatment of PF-06764427 @ 10 mg/kg

			PF-06764427 impacted corneal reflex, pinna reflex, salivation & motor activity					
Davoren et al., 2016a	C57BL/6J mice	M_1 PAM (PF-06764427): 1, 3.2, 10 mg/kg (sc)	None reported	1. Locomotion	Amphetamine-induced hyperactivity	1 per dose	Locomotion	Partial rescue of amphetamine-induced hyperactivity @ 3.2 & 10 mg/kg
Davoren et al., 2016b	Task 1: C57BL/6J mice (m; 6-8 w.o.) Task 2: Wistar rats (m) Task 3: Sprague-Dawley rats *Sprague-Dawley rats (m/f; 7-10 w.o.) & Beagle dogs (m) were used for adverse effects testing	M_1 PAM (PF-06767832): 0.32, 1, 3.2 mg/kg (mice: sc; rats: po)	Rats: increased food intake and body weight gain @ 10, 15 & 30 mg/kg. Dogs: loose stool, emesis & salivation @ 3 mg/kg and higher doses with convulsions @ 45 mg/kg	1. Locomotion	Amphetamine-induced hyperactivity	1 per dose	Locomotion	Partial rescue of amphetamine-induced hyperactivity @ 1 & 3.2 mg/kg.
				2. Morris water maze	Scop.	1 per dose	Learning and memory	Partial rescue @ 0.32 mg/kg
				3. Pre-pulse inhibition	Amphetamine disruption	1 per dose	Sensori-motor/vigilance	Partial rescue @ 1 mg/kg
Moran et al., 2018	C57BL6/J mice (m)	M_1 PAM (VU0453595): 0.3, 1, 3, 10 mg/kg (po) M_1 PAM (MK-7622): 1, 3, 10 mg/kg (po)	Convulsions induced @ 30 & 100 mg/kg MK-7622. None reported for VU0453595	1. Novel object recognition task	None	1 per dose	Learning and memory	Enhanced recognition memory @ 1, 3, 10 mg/kg
Davoren et al., 2017	Task 1: C57BL/6J mice (m; 6-8 w.o.) Task 2: Wistar rats (m)	M_1 PAM (PF-06827443): 0.32, 1, 3.2 mg/kg (sc)	Rats: soft feces @ 15 mg/kg & convulsions @ 45 mg/kg (po).	1. Locomotion	Amphetamine-induced hyperactivity	1 per dose	Locomotion	Partial rescue of amphetamine-induced hyperactivity @ all doses.
				2. Morris water maze	Scop.	5 per dose	Learning and memory	Partial rescue at 0.32 mg/kg and full rescue at 1 and 3.2 mg/kg

Abd-Elrahman et al., 2022	APPswe mice (f; 9 m.o.) *control: B6C3F1/J mice (f; 9 m.o.)	M_1 PAM (VU0486846): 10 mg/kg	None reported	1. Novel object recognition task	Mice with elevated beta amyloid	Daily dosing for 4 or 8 weeks	Learning and memory	Partial rescue with 4 week exposure. Enhancement of scores with 8 week exposure (*worse score for WT mice; WT vehicle mice performed worse @ 8 week relative to 4 week)
				2. Morris water maze + reversal morris water maze	Mice with elevated beta amyloid	Daily dosing for 4 or 8 weeks	Learning and memory; cognitive flexibility (exec. functioning)	Partial rescue for both 4 & 8 week exposure regimes.
				3. Open field test	Mice with elevated beta amyloid	Daily dosing for 4 or 8 weeks	Anxiety-like behavior (locomotion)	Partial to full rescue of anxiety-like behavior for both 4 & 8 week exposure regimes.
Sako et al., 2019	Sprague-Dawley rats (m) & Long Evans rats (m) *C57BL/6J mice were also used for identifying adverse effects	M_1 PAM (TAK-071): 0.03, 0.1, 0.3, 1, 3 mg/kg (po) M_1 PAM (T-662): 0.03, 0.1, 0.3 mg/kg (po); Controls: AChE-I (Donepezil): 0.1, 0.3, 1, 3 mg/kg (po) AChE-I (Rivastigmine): 0.1, 0.3, 1 mg/kg (ip)	Rats: Loose stool, salivation, miosis & fasciculation in rats induced by donepezil @ 10 mg/kg. Lacrimation, salivation, miosis & fasciculation induced by rivastigmine @ ≥ 3 mg/kg. Loose stool induced by TAK-071 @ 3 mg/kg and T-662 @ 0.1 mg/kg Mice: Diarrhea induced with TAK-071 @ 3 mg/kg & T-662 @ 10 mg/kg.	1. Novel object recognition task	Scop.	1 per dose	Learning and memory	Partial rescue by donepezil @ 0.3 & 1 mg/kg. Full rescue by rivastigmine @ 0.3 & 1 mg/kg. Full rescue by TAK-071 @ 0.3, 1 & 3 mg/kg. Full rescue by T-662 @ 0.1 & 0.3 mg/kg.
Ghoshal et al., 2016	C57BL6/J mice (m; 8-9 w.o.)	M_1 PAM (VU0453595): 1, 3, 10 mg/kg (ip)	None reported	1. Novel object recognition task	PCP challenge	1 per dose	Learning and memory	Partial rescue by VU0453595 @ 1, 3 & 10 mg/kg
				2. Social interaction assay	PCP challenge	1 per dose	Social interaction	Full rescue by VU0453595 @ 1, 3 & 10 mg/kg

Shirey et al., 2009	Tg2576 mice (m/f; 10-12 w.o.)	M_1 PAM (BQCA): 30 mg/kg (sc)	None reported	1. Reversal learning digging task	Mice with elevated beta amyloid	2 per dose *received compound twice but only 1 pre-reversal & 1 post-reversal measure	Cognitive flexibility (exec. functioning)	Full rescue of reversal performance by BQCA. Enhanced performance beyond just reversal (relative to even WT mice) for the initial discrimination
Fisher et al., 2016	Task 1: Wistar rats (3 m.o.) Task 2: 3xTg-AD mice (f; 12 m.o.)	M_1 PAM (AF710B): Task 1: 1, 3, 10, 30, 100 ug/kg (po) Task 2: 10 ug/kg (ip)	None reported	1. Passive avoidance	Trihexyphenidyl challenge	1 per dose	Learning and memory	Partial rescue with AF710B @ 1, 3, 10 & 30 ug/kg (po)
				2. Morris water maze	Mice with frontotemporal dementia mutation (tau mutation)	Daily dosing for 2 months (from 10-12 m.o.)	Learning and memory	Partial rescue with AF710B @ 10 ug/kg (ip)
Grannan et al., 2016	C57BL/6J mice (8-10 w.o.)	M_1 PAM (VU6004256): 1, 3, 10 mg/kg (ip)	None reported	1. Novel object recognition task	NMDA receptor subunit (NR1) knock-down mice	1 per dose	Learning and memory	Enhancement in novel object recognition of WT mice @ 10mg/kg. Full rescue @ 3 and 10 mg/kg.
				2. Cue-mediated fear conditioning task	NMDA receptor subunit (NR1) knock-down mice	1 per dose	Learning and memory	Partial rescue @ 10 mg/kg.
				3. Spontaneous locomotor activity	NMDA receptor subunit (NR1) knock-down mice	1 per dose	Locomotion	Full rescue @ 1 & 10 mg/kg
Maksymetz et al., 2019	C57BL/6J mice	M_1 antagonist (VU0255035): 3, 10, 30 mg/kg (ip) M_1 PAM (VU0453595): 10 mg/kg (ip)	None reported	1. Contextual fear conditioning	None	2 per dose (VU0255035)	Learning and memory	Significantly impaired contextual extinction with VU0255035 @ 30 mg/kg
				2. Stress-enhanced fear learning	None	1 per dose (VU0453595)	Learning and memory	Enhanced extinction with VU0453595 @ 10 mg/kg
Smith et al., 2022	Mecp2 hetero- and homozygote mice (f; 20 w.o.)	M_1 PAM (VU0453595): 10 mg/kg (ip)	None reported	1. Open field test	Mecp2 heterozygotes	1 per dose	Anxiety-like behavior (locomotion)	No effect
				2. 3-chamber social preference assay	Mecp2 heterozygotes	1 per dose	Social recognition and memory	Rescue of biased exploration towards new mice

				3. Novel object recognition assay	Mecp2 heterozygotes	1 per dose	Learning and memory	Full rescue of novel object preference
				4. Contextual fear conditioning	Mecp2 heterozygotes	1 per dose	Learning and memory	Full rescue of freezing behavior
Walker et al., 2022	6-8 (per group) Indiana alcohol-preferring rats (m; 8 w.o.)	M_1 PAM (PF-06767832): 1 mg/kg (ip)	None reported	1. Locomotion	None (alcohol preferring rats only)	1 per dose	Locomotion	No effect
				2. Alcohol self-administration	None (alcohol preferring rats only)	2 per dose (1 per measure)	Alcohol self-administration; motivation	Reduced alcohol self-administration without altering motivation for alcohol
				3. Sucrose self-administration	None (alcohol preferring rats only)	2 per dose (1 per measure) *same mice used across assays 2 & 3	Food and water self-administration	Reduced sucrose self-administration, as well as food and water consumption
Kurimoto et al., 2021	C57BL/6 mice (11-18 w.o.)	M_1 PAM (TAK-071): 0.03, 0.1, 0.3 mg/kg (po) M_1 PAM (MK-7622): 1, 3, 10 mg/kg (ip) *NO positive results and only used in task 1	None reported	1. Social approach-avoidance test	A: Schizophrenia mice model (miR-137 Tg) B: maternal exposure to poly I:C (reported to induce Schizophrenia symptoms) C: haloperidol challenge	1 per dose	Sociability	B: Full rescue of social sniffing @ 0.1 & 0.3 mg/kg
				2. Y-maze task		1 per dose	Working memory	A: Full rescue of alternations @ 0.3 mg/kg B: Full rescue of alternations @ 0.1 mg/kg A/C: Full rescue of haloperidol-induced reduction in alternations in miR-137 Tg mice.
				3. Novel object recognition task		1 per dose	Learning and memory	A: full rescue @ 0.1 & 0.3 mg/kg
				4. Pre-pulse inhibition test		1 per dose	Sensori-motor/vigilance	A: full rescue @ 0.3 mg/kg
Kucinski et al., 2021	Sprague-Dawley rats (f; 2-3 m.o.)	M_1 PAM (TAK-071): 0.1, 0.3 mg/kg (ig)	None reported	1. Michigan complex movement control task	Rats with dual cholinergic-dopamine loss	7 per dose (1 daily)	Complex motor control	Partial rescue of complex motor control @ both 0.1 (best) & 0.3 mg/kg
Mandai et al., 2020	Long Evans rats (7 w.o.) *Sprague-Dawley rats were used for	AChE-I (Donepezil): 0.1, 1 mg/kg (po) M_1 PAM (MK-7622): 1, 3, 10 mg/kg (po)	T-495 induced diarrhea and in 1/6 rats, convulsions +	1. Novel object recognition task	Scop.	1 dose per regime (both PAMs alone or combined with donepezil)	Learning and memory	Rescue by T-495 @ 1 & 3 mg/kg. Partial rescue by MK-7622 @ 3 & 10 mg/kg. Rescue by donepezil (0.1 mg/kg) & T-495 (0.3 mg/kg).

	characterization of aversive side effects	M_1 PAM (T-495): 0.3, 1, 3 mg/kg (po)	salivation @ 100 mg/kg. MK-7622 induced diarrhea at 3 mg/kg	2. Contextual fear conditioning	Mouse model of dementia and Parkinson's (CaMKIIα-tTA/A543T α-syn dTg)	2 per doses (donepezil or T-495)	Learning and memory	Partial rescue by donepezil @ 1 mg/kg. Partial rescue by T-495 @ 3 mg/kg.
				3. Y-maze	Mouse model of dementia and Parkinson's (CaMKIIα-tTA/A543T α-syn dTg)	1 per dose (donepezil or T-495)	Working memory	Full rescue by donepezil @ 1 mg/kg. Partial rescue by T-495 @ 3 mg/kg.
Kucinski et al., 2020	3-4 (per group) Sprague-Dawley rats (m/f; 2-3 m.o.)	M_1 PAM (TAK-071): 0.1, 0.3 mg/kg (ig)	None reported	1. Sustained attention task	A: signal duration<br>B: Cholinergic lesioned rodents<br>C: distractor (flashing light)	6 per dose (1 daily)	Attention/vigilance	A/B: no effect<br>A/C: no effect<br>A/Post C (after termination of distractor): enhanced performance for lesioned and non-lesioned mice (strongest effect on lesioned) @ 0.1 mg/kg
Choy et al., 2016	C57Bl/6J (m; 2-4 m.o.)	M_1 PAM (BQCA): 1, 3, 5, 10, 20 mg/kg (sc or ip); combined with anti-psychotic drugs but only considered here when used alone	None reported for BQCA	1. Pre-pulse inhibition	MK-801 challenge	1 session per dose (3 pulses)	Sensori-motor/vigilance	No effect with BQCA alone
				2. Y-maze	MK-801 challenge	1 per dose	Working memory	No effect with BQCA alone
Total studies utilizing PAMs: 28	Rodents used:<br>-Sprague-Dawley rats: 9/28 studies<br>-C57BL mice: 13/28 studies<br>-Long Evans rats/Wistar rats/ other rodents: 13/28 studies	M_1 PAMs:<br>-VU compounds: 9/28 studies<br>-BQCA/PQCA: 9/28 studies<br>-PF compounds: 6/28 studies<br>-TAK-071: 4/28 studies<br>-MK-7622: 3/28 studies	Reported for several PAMs and other cholinergic agents	1/28 tasks had no behavioral component<br>14/28 had 1 only 1 task<br>6/28 had 2 tasks<br>5/28 had 3 tasks<br>2/28 had 4 tasks	-Scopolamine challenge: 7/28 studies<br>-Other compound challenges (amphetamine, PCP, alcohol etc): 9/28 studies<br>-Aged rodents/ genetic model	Average number of determinations: 1 (vast majority used only 1 determination)<br>-4/28 studies contained some daily dosing regime<br>-5/28 studies contained more	Cognitive domains tested:<br>-Learning and memory (19/28; 5 of those had more than 1 task in this domain)<br>-Locomotion/ motor control (9/28)<br>-Working memory (6/28)	Some behavioral enhancement (usually a rescue) in almost all studies with at least 1 dose of M_1 PAMs

		-Other: 5/27 studies			of disease: 7/28 studies<br>-Behavioral: 3/28 studies<br>-Other (prions, lesions): 4/28 studies<br>-None: 3/28 studies	than 1 determination in at least 1 dose (non-daily dosing)	-Attention/ vigilance (3/28)<br>-Social behavior (3/28)<br>-Cognitive flexibility/exec. function (2/28)<br>-Satiety/drug abuse (1/28)	

References

1. T. Womelsdorf, *et al.*, A Kiosk Station for the Assessment of Multiple Cognitive Domains and Enrichment of Monkeys. *Front Behav Neurosci* **15**, 721069 (2021).
2. M. R. Watson, B. Voloh, C. Thomas, A. Hasan, T. Womelsdorf, USE: An integrative suite for temporally-precise psychophysical experiments in virtual environments for human, nonhuman, and artificially intelligent agents. *J Neurosci Methods* **326** (2019).
3. S. A. Hassani, *et al.*, Dose-Dependent Dissociation of Pro-cognitive Effects of Donepezil on Attention and Cognitive Flexibility in Rhesus Monkeys. *Biological Psychiatry Global Open Science* **3**, 68–77 (2021).
4. R. W. Gould, *et al.*, Modulation of arousal and sleep/wake architecture by M1 PAM VU0453595 across young and aged rodents and nonhuman primates. *Neuropsychopharmacology* **45**, 2219–2228 (2020).
5. E. Kurimoto, M. Nakashima, H. Kimura, M. Suzuki, TAK-071, a muscarinic M1 receptor positive allosteric modulator, attenuates scopolamine-induced quantitative electroencephalogram power spectral changes in cynomolgus monkeys. *PLoS One* **14**, 1–15 (2019).
6. J. M. Uslaner, *et al.*, Preclinical to human translational pharmacology of the novel M1positive allosteric modulator MK-7622. *Journal of Pharmacology and Experimental Therapeutics* **365**, 556–566 (2018).
7. J. M. Uslaner, *et al.*, The muscarinic M1 receptor positive allosteric modulator PQCA improves cognitive measures in rat, cynomolgus macaque, and rhesus macaque. *Psychopharmacology (Berl)* **225**, 21–30 (2013).
8. H. S. Lange, C. E. Cannon, J. T. Drott, S. D. Kuduk, J. M. Uslaner, The M1 muscarinic positive allosteric modulator PQCA improves performance on translatable tests of memory and attention in rhesus monkeys. *Journal of Pharmacology and Experimental Therapeutics* **355**, 442–450 (2015).
9. J. D. Vardigan, *et al.*, Improved cognition without adverse effects: Novel M1 muscarinic potentiator compares favorably to donepezil and xanomeline in rhesus monkey. *Psychopharmacology (Berl)* **232**, 1859–1866 (2015).
10. C. Chambon, N. Wegener, A. Gravius, W. Danysz, A new automated method to assess the rat recognition memory: Validation of the method. *Behavioural Brain Research* **222**, 151–157 (2011).
11. C. Chambon, C. Jatzke, N. Wegener, A. Gravius, W. Danysz, Using cholinergic M1 receptor positive allosteric modulators to improve memory via enhancement of brain cholinergic communication. *Eur J Pharmacol* **697**, 73–80 (2012).
12. S. J. Bradley, *et al.*, M1 muscarinic allosteric modulators slow prion neurodegeneration and restore memory loss. *Journal of Clinical Investigation* **127**, 487–499 (2017).
13. L. Dwomoh, *et al.*, M_1 muscarinic receptor activation reduces the molecular pathology and slows the progression of prion-mediated neurodegenerative disease. *Sci Signal* **15** (2022).
14. J. M. Rook, *et al.*, A Novel M 1 PAM VU0486846 Exerts Efficacy in Cognition Models without Displaying Agonist Activity or Cholinergic Toxicity. *ACS Chem Neurosci* **9**, 2274–2285 (2018).

15. J. M. Rook, *et al.*, Diverse Effects on M1 Signaling and Adverse Effect Liability within a Series of M1 Ago-PAMs. *ACS Chem Neurosci* **8**, 866–883 (2017).
16. L. Ma, *et al.*, Selective activation of the M1 muscarinic acetylcholine receptor achieved by allosteric potentiation. *Proc Natl Acad Sci U S A* **106**, 15950–15955 (2009).
17. V. Puri, X. Wang, J. D. Vardigan, S. D. Kuduk, J. M. Uslaner, The selective positive allosteric M1 muscarinic receptor modulator PQCA attenuates learning and memory deficits in the Tg2576 Alzheimer's disease mouse model. *Behavioural Brain Research* **287**, 96–99 (2015).
18. J. E. Davoren, *et al.*, Design and optimization of selective azaindole amide M1 positive allosteric modulators. *Bioorg Med Chem Lett* **26**, 650–655 (2016).
19. J. E. Davoren, *et al.*, Discovery of the Potent and Selective M1 PAM-Agonist N-[(3R,4S)-3-Hydroxytetrahydro-2H-pyran-4-yl]-5-methyl-4-[4-(1,3-thiazol-4-yl)benzyl]pyridine-2-carboxamide (PF-06767832): Evaluation of Efficacy and Cholinergic Side Effects. *J Med Chem* **59**, 6313–6328 (2016).
20. J. E. Davoren, *et al.*, Design and Synthesis of γ- And δ-Lactam M1 Positive Allosteric Modulators (PAMs): Convulsion and Cholinergic Toxicity of an M1-Selective PAM with Weak Agonist Activity. *J Med Chem* **60**, 6649–6663 (2017).
21. S. P. Moran, *et al.*, M1-positive allosteric modulators lacking agonist activity provide the optimal profile for enhancing cognition. *Neuropsychopharmacology* **43**, 1763–1771 (2018).
22. K. S. Abd-Elrahman, S. Sarasija, T. L. Colson, S. S. G. Ferguson, A positive allosteric modulator for the muscarinic receptor (M1 mAChR) improves pathology and cognitive deficits in female <scp>APPswe</scp> /PSEN1ΔE9 mice. *Br J Pharmacol* **179**, 1769–1783 (2022).
23. Y. Sako, *et al.*, TAK-071, a novel M1 positive allosteric modulator with low cooperativity, improves cognitive function in rodents with few cholinergic side effects. *Neuropsychopharmacology* **44**, 950–960 (2019).
24. A. Ghoshal, *et al.*, Potentiation of M1 muscarinic receptor reverses plasticity deficits and negative and cognitive symptoms in a schizophrenia mouse model. *Neuropsychopharmacology* **41**, 598–610 (2016).
25. J. K. Shirey, *et al.*, A selective allosteric potentiator of the M1 muscarinic acetylcholine receptor increases activity of medial prefrontal cortical neurons and restores impairments in reversal learning. *Journal of Neuroscience* **29**, 14271–14286 (2009).
26. A. Fisher, *et al.*, AF710B, a novel M1/σ1 agonist with therapeutic efficacy in animal models of Alzheimer's disease. *Neurodegener Dis* **16**, 95–110 (2016).
27. M. D. Grannan, *et al.*, Prefrontal Cortex-Mediated Impairments in a Genetic Model of NMDA Receptor Hypofunction Are Reversed by the Novel M1 PAM VU6004256. *ACS Chem Neurosci* **7**, 1706–1716 (2016).
28. J. Maksymetz, *et al.*, M1 Muscarinic Receptors Modulate Fear-Related Inputs to the Prefrontal Cortex: Implications for Novel Treatments of Posttraumatic Stress Disorder. *Biol Psychiatry* **85**, 989–1000 (2019).
29. M. Smith, *et al.*, Clinical and Preclinical Evidence for M1 Muscarinic Acetylcholine Receptor Potentiation as a Therapeutic Approach for Rett Syndrome. *Neurotherapeutics* **19**, 1340–1352 (2022).

30. L. C. Walker, *et al.*, M1 muscarinic receptor activation decreases alcohol consumption via a reduction in consummatory behavior. *Pharmacol Res Perspect* **10**, 1–10 (2022).
31. E. Kurimoto, R. Yamada, T. Hirakawa, H. Kimura, Therapeutic potential of TAK-071, a muscarinic M1 receptor positive allosteric modulator with low cooperativity, for the treatment of cognitive deficits and negative symptoms associated with schizophrenia. *Neurosci Lett* **764**, 136240 (2021).
32. A. Kucinski, M. Sarter, Reduction of falls in a rat model of PD falls by the M1 PAM TAK-071. *Psychopharmacology (Berl)* **238**, 1953–1964 (2021).
33. A. Kucinski, K. B. Phillips, A. Koshy Cherian, M. Sarter, Rescuing the attentional performance of rats with cholinergic losses by the M1 positive allosteric modulator TAK-071. *Psychopharmacology (Berl)* **237**, 137–153 (2020).
34. T. Mandai, *et al.*, T-495, a novel low cooperative M1 receptor positive allosteric modulator, improves memory deficits associated with cholinergic dysfunction and is characterized by low gastrointestinal side effect risk. *Pharmacol Res Perspect* **8**, 1–17 (2020).
35. K. H. C. Choy, *et al.*, Positive allosteric modulation of the muscarinic M1 receptor improves efficacy of antipsychotics in mouse glutamatergic deficit models of behavior. *Journal of Pharmacology and Experimental Therapeutics* **359**, 354–365 (2016).
36. K. S. Abd-Elrahman, S. Sarasija, T. L. Colson, S. S. G. Ferguson, A positive allosteric modulator for the muscarinic receptor (M1 mAChR) improves pathology and cognitive deficits in female <scp>APPswe</scp> /PSEN1ΔE9 mice. *Br J Pharmacol* **179**, 1769–1783 (2022).
37. S. D. Glick, M. E. Jarvik, Differential effects of amphetamine and scopolamine on matching performance of monkeys with lateral frontal lesions. *J Comp Physiol Psychol* **73**, 307–313 (1970).
38. R. T. Bartus, H. R. Johnson, Short-term memory in the rhesus monkey: Disruption from the anti-cholinergic scopolamine. *Pharmacol Biochem Behav* **5**, 39–46 (1976).
39. J. J. Buccafusco, A. V. Terry, Donepezil-induced improvement in delayed matching accuracy by young and old rhesus monkeys. *Journal of Molecular Neuroscience* **24**, 85–91 (2004).
40. J. J. Buccafusco, W. J. Jackson, J. D. Stone, A. V. Terry, Sex dimorphisms in the cognitive-enhancing action of the Alzheimer's drug donepezil in aged Rhesus monkeys. *Neuropharmacology* **44**, 381–389 (2003).
41. J. J. Buccafusco, *et al.*, The scopolamine-reversal paradigm in rats and monkeys: The importance of computer-assisted operant-conditioning memory tasks for screening drug candidates. *Psychopharmacology (Berl)* **199**, 481–494 (2008).
42. N. M. J. Rupniak, S. J. Tye, M. J. Field, Enhanced performance of spatial and visual recognition memory tasks by the selective acetylcholinesterase inhibitor E2020 in rhesus monkeys. *Psychopharmacology (Berl)* **131**, 406–410 (1997).
43. A. W. C. Oliveira, *et al.*, Scopolamine and MK-801 impair recognition memory in a new spontaneous object exploration task in monkeys. *Pharmacol Biochem Behav* **211**, 1–6 (2021).
44. H. Tsukada, *et al.*, Effects of Acute Acetylcholinesterase Inhibition on the Cerebral Cholinergic Neuronal System and Cognitive Function: Functional Imaging of the

Conscious Monkey Brain Using Animal PET in Combination with Microdialysis. *Synapse* **52**, 1–10 (2004).
45. M. A. Taffe, M. R. Weed, L. H. Gold, Scopolamine alters rhesus monkey performance on a novel neuropsychological test battery. *Cognitive Brain Research* **8**, 203–212 (1999).
46. B. Knakker, *et al.*, Delay-dependent cholinergic modulation of visual short-term memory in rhesus macaques. *Behavioural Brain Research* **396**, 112897 (2021).
47. M. J. Callahan, Combining tacrine with milameline reverses a scopolamine-induced impairment of continuous performance in rhesus monkeys. *Psychopharmacology (Berl)* **144**, 234–238 (1999).
48. P. M. Callahan, E. J. Hutchings, N. J. Kille, J. M. Chapman, A. V. Terry, Positive allosteric modulator of alpha 7 nicotinic-acetylcholine receptors, PNU-120596 augments the effects of donepezil on learning and memory in aged rodents and non-human primates. *Neuropharmacology* **67**, 201–212 (2013).
49. E. P. Lebois, *et al.*, Disease-Modifying Effects of M1 Muscarinic Acetylcholine Receptor Activation in an Alzheimer's Disease Mouse Model. *ACS Chem Neurosci* **8**, 1177–1187 (2017).
50. G. J. Digby, *et al.*, Novel allosteric agonists of M 1 muscarinic acetylcholine receptors induce brain region-specific responses that correspond with behavioral effects in animal models. *Journal of Neuroscience* **32**, 8532–8544 (2012).
51. C. H. Xiong, *et al.*, M1 muscarinic receptors facilitate hippocampus-dependent cognitive flexibility via modulating GluA2 subunit of AMPA receptors. *Neuropharmacology* **146**, 242–251 (2019).
52. M. R. Weed, *et al.*, Performance norms for a rhesus monkey neuropsychological testing battery: Acquisition and long-term performance. *Cognitive Brain Research* **8**, 185–201 (1999).
53. R. G. Wither, S. E. Boehnke, A. Lablans, B. Armitage-Brown, D. P. Munoz, Behavioral shaping of rhesus macaques using the Cambridge neuropsychological automated testing battery. *J Neurosci Methods* **342**, 108803 (2020).
54. D. Palmer, *et al.*, Touchscreen cognitive testing: Cross-species translation and co-clinical trials in neurodegenerative and neuropsychiatric disease. *Neurobiol Learn Mem* **182**, 107443 (2021).
55. J. R. Ellis, *et al.*, Muscarinic and nicotinic receptors synergistically modulate working memory and attention in humans. *International Journal of Neuropsychopharmacology* **9**, 175–189 (2006).
56. T. Edginton, J. M. Rusted, Separate and combined effects of scopolamine and nicotine on retrieval-induced forgetting. *Psychopharmacology (Berl)* **170**, 351–357 (2003).
57. P. Broks, *et al.*, Modelling dementia: Effects of scopolamine on memory and attention. *Neuropsychologia* **26**, 685–700 (1988).
58. H. S. Lange, C. E. Cannon, J. T. Drott, S. D. Kuduk, J. M. Uslaner, The M1 muscarinic positive allosteric modulator PQCA improves performance on translatable tests of memory and attention in rhesus monkeys. *Journal of Pharmacology and Experimental Therapeutics* **355**, 442–450 (2015).

59. M. A. Taffe, M. R. Weed, T. Gutierrez, S. A. Davis, L. H. Gold, Differential muscarinic and NMDA contributions to visuo-spatial paired-associate learning in rhesus monkeys. *Psychopharmacology (Berl)* **160**, 253–262 (2002).
